# Gentle Label‐Free Nonlinear Optical Imaging Relaxes Linear‐Absorption‐Mediated Triplet

**DOI:** 10.1002/advs.202415648

**Published:** 2025-05-28

**Authors:** Geng Wang, Lianhuang Li, Janet E. Sorrells, Jianxin Chen, Haohua Tu

**Affiliations:** ^1^ Department of Electrical and Computer Engineering University of Illinois at Urbana‐Champaign Urbana IL 61801 USA; ^2^ Beckman Institute for Advanced Science and Technology University of Illinois at Urbana‐Champaign Urbana IL 61801 USA; ^3^ Key Laboratory of OptoElectronic Science and Technology for Medicine of Ministry of Education Fujian Provincial Key Laboratory of Photonics Technology Fujian Normal University Fuzhou 350007 China

**Keywords:** fluorescence microscopy, phototoxicity, nonlinear optical imaging, triplet

## Abstract

Sample health is critical for live‐cell fluorescence microscopy and has promoted light‐sheet microscopy that restricts its ultraviolet–visible excitation to one plane inside a 3D sample. It is thus intriguing that laser‐scanning nonlinear optical microscopy, which similarly restricts its near‐infrared excitation, has not broadly enabled gentle label‐free molecular imaging. It is hypothesized that intense near‐infrared excitation induces phototoxicity via linear absorption of intrinsic biomolecules with subsequent triplet buildup, rather than the commonly assumed mechanism of nonlinear absorption. Using a reproducible phototoxicity assay based on the time‐lapse elevation of autofluorescence (hyper‐fluorescence) from a homogeneous tissue model (chicken breast), strong evidence is provided supporting this hypothesis. The study justifies a simple imaging technique, e.g., rapidly scanned sub‐80‐fs excitation with full triplet‐relaxation, to mitigate this ubiquitous linear‐absorption‐mediated phototoxicity independent of sample types. The corresponding label‐free imaging can track freely moving *C. elegans* in real‐time at an irradiance up to one‐half of water optical breakdown.

## Graphical abstract

The phototoxicity in nonlinear optical microscopy arises mainly from linear absorption of intrinsic biomolecules rather than nonlinear absorption. This mechanism is supported by a reproducible phototoxicity assay based on widely available chicken breast. A simple imaging strategy with short rapidly scanned femtosecond pulses mitigates this damage, enabling real‐time label‐free imaging of *C. elegans* and live tissue at high irradiance.

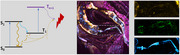

## 1 Introduction

**Figure 1:**
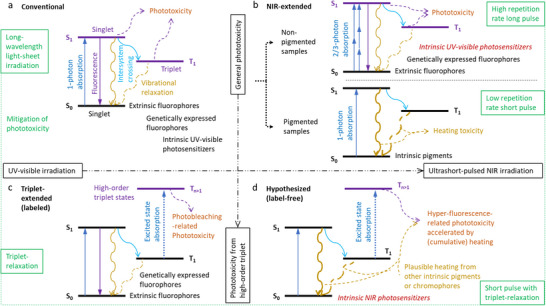
Mechanisms of phototoxicity based on Jablonski diagram. a) Conventional mechanism with UV–visible excitation. b) NIR‐extended mechanism with scanning ultrashort‐pulsed NIR excitation at the focus of a microscope objective. c) Triplet‐extended mechanism with UV–visible excitation of labeling fluorophores. d) Hypothesized mechanism at phototoxicity threshold with the same NIR excitation but without the labeling. One‐photon absorption of certain ubiquitous chromophore followed by efficient inter‐crossing to a first‐order triplet state results in high‐order triplet‐induced heating‐accelerated white hyper‐fluorescence (WHF).

Due to plausible artifacts from light itself,^[^ ^1^ ^]^ live‐cell fluorescence imaging has increasingly emphasized sample health over metrics, such as signal‐to‐noise ratio and spatiotemporal resolution.^[^ ^2^ ^]^ The conventional mechanism of phototoxicity attributes the toxicity to the excited singlet/triplet states of extrinsic (labeling) UV–visible‐absorbing fluorophores^[^ ^2^, ^3^ ^]^ (**Figure** **1**a). Thus, wide‐field planar excitation that restricts the excitation to a focal plane (e.g., light‐sheet microscopy) has gained popularity to avoid out‐of‐focus phototoxicity in standard wide‐field or laser‐scanning fluorescence microscopy.^[^ ^3^, ^4^ ^]^ Another technique is to excite the labeling fluorophores at the long‐wavelength end of UV–visible excitation (300–650 nm), which mitigates the phototoxicity from intrinsic photosensitizers^[^ ^5^ ^]^ (Figure 1a) but limits the choices to fluorescently label the sample. By avoiding this critical limitation from popular continuous‐wavelength excitation, near‐infrared (NIR) extension (700–1300 nm) with laser‐scanning ultrashort (<10 ps) pulses has recovered the planar excitation and efficiently excited the extrinsic fluorophores via nonlinear absorption^[^ ^6^ ^]^ (Figure 1b).

However, raster scanned ultrashort‐pulsed NIR excitation at the focus of a high numerical aperture (NA) microscope objective has either facilitated linear‐absorption‐mediated heating toxicity in pigmented samples, which should be mitigated by low repetition rate short pulse,^[^ ^7^ ^]^ or commonly assumed nonlinear‐absorption‐mediated phototoxicity in nonpigmented samples,^[^ ^8^, ^9^ ^]^ which should be mitigated by high repetition rate long pulse.^[^ ^10^, ^11^ ^]^ Because the two are differentiated rather arbitrarily based on often unavailable NIR absorption property of the sample,^[^ ^12^ ^]^ no universal mitigation technique exists for both cases (Figure 1b). Also, the related NIR‐extended mechanism favors a light‐dose (fluence) threshold for phototoxicity^[^ ^9^, ^13^ ^]^ over an irradiance threshold (typically proportional to average power or pulse energy), which is inconsistent with empirical experiences^[^ ^8^, ^14^ ^]^ (Table S1, Supporting Information). Moreover, this mechanism offers no satisfactory explanation on why the phototoxicity decreases with increased speed of fast‐axis scanning, even though other parameters are comparable (Table S2, Supporting Information).

We strive to overcome these deficiencies in knowledge based on another extension of the conventional mechanism, i.e., triplet‐extended mechanism of photobleaching‐related phototoxicity^[^ ^2^, ^15^ ^]^ from the labeling agents of extrinsic fluorophores and genetically expressed fluorophores^[^ ^16^ ^]^ (Figure 1c). This mechanism offers a satisfactory explanation (i.e., triplet‐relaxation) on why a fast‐scanning speed effectively mitigates the phototoxicity in both confocal fluorescence microscopy^[^ ^17^ ^]^ and stimulated emission depletion microscopy.^[^ ^18^, ^19^ ^]^ Specifically, high‐order triplet states produced by excited state absorption,^[^ ^16^ ^]^ rather than first‐order triplet/singlet states and high‐order singlet states, dominate the observed UV–visible phototoxicity from bioassays^[^ ^17^, ^19^ ^]^ (Figure 1c). Considering the similar dependence of the NIR phototoxicity on the scanning speed (Table S2, Supporting Information), we hypothesize that the corresponding NIR extension would follow a similar mechanism (Figure 1d) in label‐free (clinically permissible) nonlinear optical imaging (Table S3, Supporting Information). Our hypothesized mechanism asserts that linear absorption of intrinsic NIR photosensitizers mediates the NIR phototoxicity in unlabeled samples (Figure 1d), rather than the commonly assumed nonlinear absorption of intrinsic UV–visible photosensitizers^[^ ^8^ ^]^ (Figure 1b), such as NAD(P)H, flavins, and porphyrins.^[^ ^14^ ^]^ This is plausible because in a broad (nonimaging) context, the existence of intrinsic NIR photosensitizers has been demonstrated in *E. coli* inside an optical trap^[^ ^20^ ^]^ and cultured cells under phototherapy.^[^ ^21^ ^]^

## 2 Results

### 2.1 Chicken Breast Model Quantifies Homogeneous Hyper‐Fluorescence

Our study is motivated by the observation of NIR photosensitizers, such as Cytochrome C oxidase, which has heme and copper centers to absorb light throughout NIR toward 1110 nm and beyond.^[^ ^22^ ^]^ The hypothesized mechanism of high‐order triplet‐mediated phototoxicity accelerated by linear absorption‐induced heating (Figure 1d) is consistent with the heating‐accelerated phototoxicity in excessive photobiomodulation,^[^ ^23^ ^]^ which is generally applicable to pigmented samples, such as skin and retina (Figure 1b). To test this mechanism, we revisit the intrinsic indicator of phototoxicity specific to nonlinear optical microscopy, i.e., elevated autofluorescence during time‐lapse imaging of diverse (live) cell/tissue specimens.^[^ ^24^ ^]^ This effect has been observed by different groups with widely varied excitation‐scanning parameters and samples, resulting in different terminologies, such as “white flashes,” “flickering/broadband luminescence,” “fluorescent scar/lesion,” “photomodulation,” “photoenhancement,” and “hyper‐fluorescence” (Table S4, Supporting Information). Despite the established functional link to impaired cell cloning^[^ ^8^ ^]^ and apoptosis,^[^ ^25^ ^]^ these different terminologies may have hindered a general understanding of the same underlying phenomenon. Because this phenomenon has not been observed by linear optical microscopy, we treat it as a unique feature of nonlinear optical microscopy. We adopt “white hyper‐fluorescence” (WHF) throughout this paper to emphasize its broadband emission and inline (built‐in) indication of phototoxicity^[^ ^26^ ^]^ in unlabeled biological samples.

**Figure 2:**
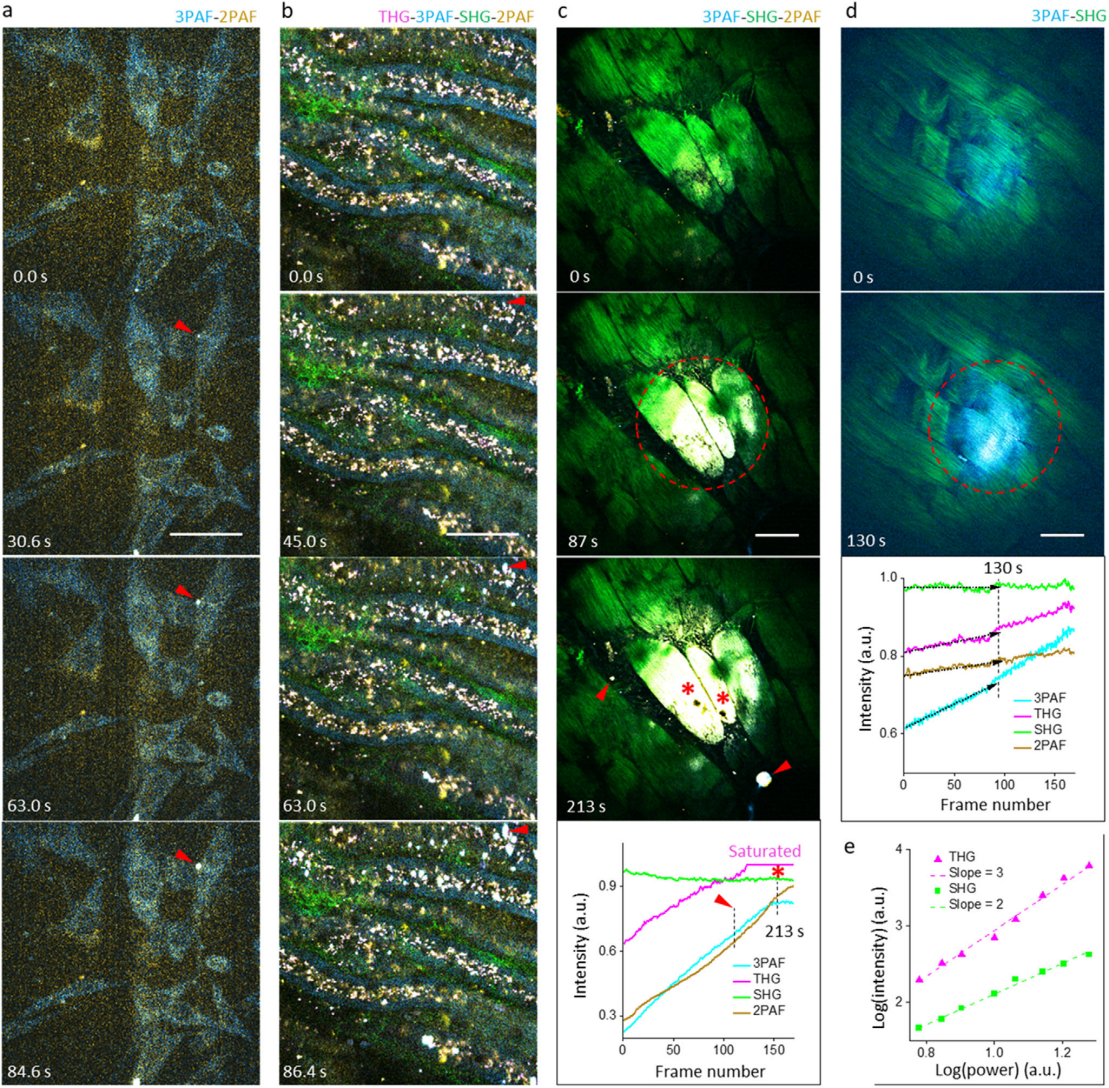
Phototoxicity observed during time‐lapse SLAM‐based imaging. a) Cultured hamster kidney cells imaged by eSLAM at a frame rate of 0.56 Hz, showing “white” heterogeneous WHF due to simultaneous increase of 3PAF/cyan and 2PAF/yellow signals (arrowheads). b) Mouse kidney tissue imaged ex vivo by eSLAM at a frame rate of 0.56 Hz, showing similar WHF (arrowheads) in one exemplary spot among several spots. c) Chicken breast imaged by pSLAM (25 mW, spatiotemporal bin‐10) at a frame rate of 0.7 Hz, showing emergence of homogeneous WHF (broken cycle) followed by the heterogeneous WHF (arrowheads) and subsequent cavitation (stars); bottom panel shows integrated signals over one frame versus frame number during time‐lapse imaging. d) Chicken breast imaged by pSLAM at a lower power (15 mW, spatiotemporal bin‐10), showing only the homogeneous WHF (broken cycle); bottom panel shows the integrated signals with initial linear growth (arrowed lines). e) Integrated THG/SHG signals versus power (baseline illumination) in different FOVs that follow power‐3/power‐2 law according to the photon order of nonlinear optical processes. Scale bar: 50 µm.

We developed a portable microscope of simultaneous label‐free autofluorescence‐multiharmonic (SLAM) microscopy (pSLAM) with flexible excitation‐scanning parameters for time‐lapse imaging of live cells (**Figure** **2**a) and ex vivo mouse kidney (Figure 2b), with four simultaneously acquired molecular contrasts (detection channels) of two‐/three‐photon‐excited autofluorescence (2PAF/3PAF) and second‐/third‐harmonic generation (SHG/THG).^[^ ^27^ ^]^ Alternatively, we incorporated the fluorescence lifetime imaging microscopy (FLIM) capability^[^ ^28^ ^]^ to build an extended version of SLAM microscope (eSLAM) with a faster imaging speed.^[^ ^29^ ^]^ The heterogeneous WHF detected by eSLAM across 2PAF‐3PAF detection spectrum of 420–640 nm (Figure 2a,b, arrowheads; and Table S5, Supporting Information) was not amenable for quantification due to its form of localized point‐spreading spots unevenly distributed in the field of view (FOV) (Videos S1 and S2, Supporting Information). In contrast, under an illumination of pSLAM (Table S6, spatiotemporal bin‐10, Supporting Information) except for a higher average power, we observed the emergence of homogeneous WHF in chicken breast tissue in the form of uniformly elevated autofluorescence across a large area of FOV (Figure 2c, broken cycle; and Video S3, Supporting Information) before the occurrence of similar heterogeneous WHF (Figure 2c, arrowheads) and subsequent cavitation (Figure 2c, stars) observed also in skin tissue at ≈110 °C temperature.^[^ ^7^ ^]^ Lowering the power avoided the heterogeneous WHF in time‐lapse imaging but retained the homogeneous WHF until a threshold at WHF onset was reached (14 mW in Figure S1a, Supporting Information). At or slightly above this threshold power, the homogeneous WHF increased linearly during the time‐lapse imaging (Figure 2d, arrowed lines). The homogeneous WHF attains a spatial distribution that approximates the illumination field measured in a fluorophore solution,^[^ ^29^ ^]^ indicating the field strength‐dependent phototoxicity. The increased THG signal most likely arises from the blue edge of WHF, while the corresponding increase in SHG signal may be canceled by a decrease due to thermal denaturing^[^ ^30^ ^]^ (Figure 2c,d, bottom panels).

To test whether homogeneous WHF can be induced in live cells rather than tissue samples, we revisited the “photo‐enhancement” effect observed from rabbit red blood cells (Table S4, Supporting Information) and confirmed the applicability of the homogeneous WHF to live cells (Video S4, Supporting Information). In contrast to the heterogeneous WHF and cavitation, the homogeneous WHF is more suitable for quantification due to uniform morphology, independence on dosage, and linear growth at an early stage of phototoxicity. Under another illumination (Table S6, baseline, Supporting Information) except for a variable power, different FOVs in one sample of chicken breast at one controlled imaging depth (15±5 µm fixed) largely follows the power laws of nonlinear signals, with an error of <20% (Figure 2e). We therefore selected chicken breast (over the red blood cells) as a readily available model to reproducibly quantify the homogeneous WHF under different illuminations.

### 2.2 Linear Rather Than Nonlinear Absorption Induces Hyper‐Fluorescence

**Figure 3:**
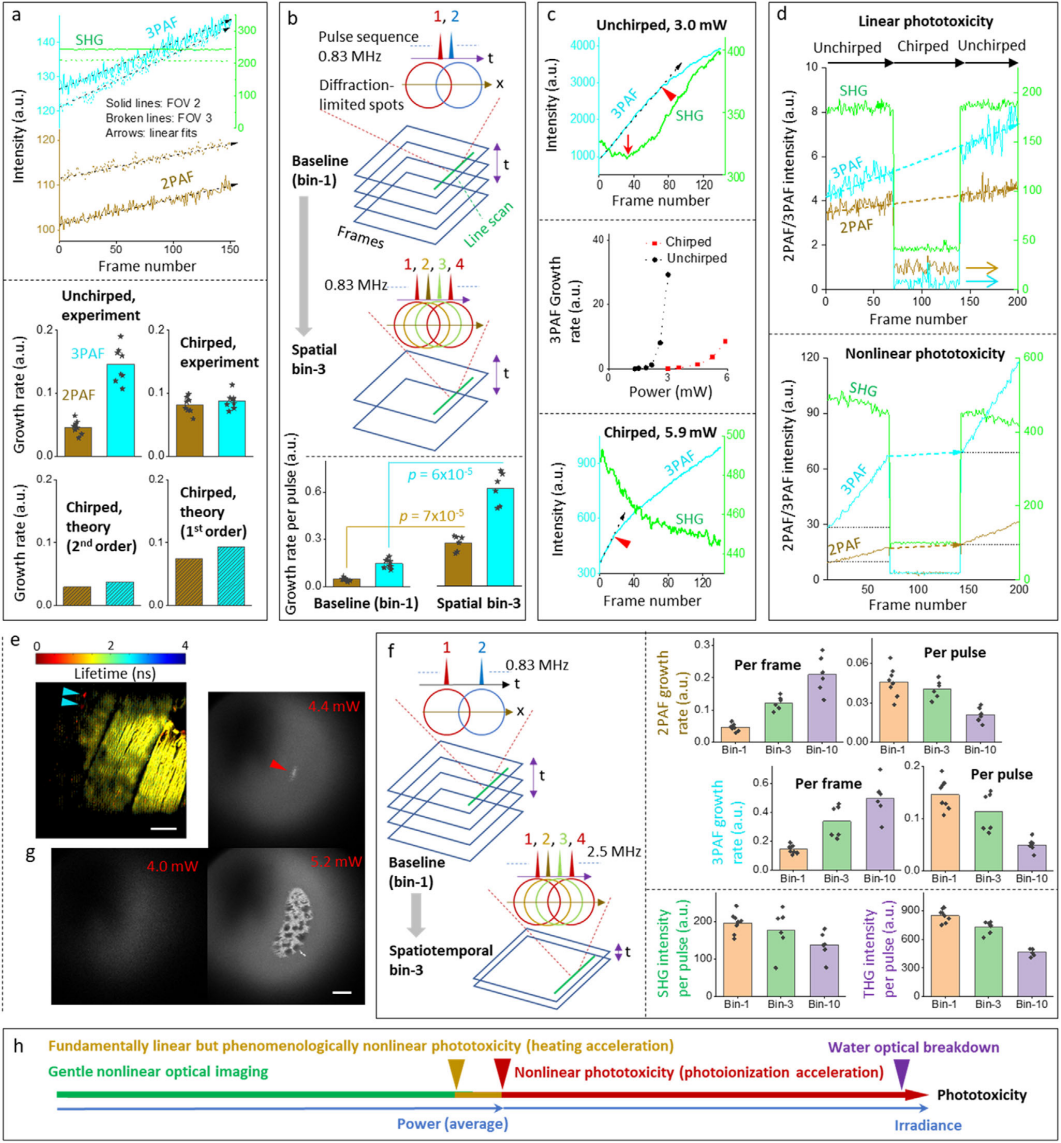
Characteristic features of homogeneous WHF‐revealed phototoxicity. a) (Top) Reproducible 2PAF and 3PAF growth rates under the baseline illumination of time‐lapse pSLAM imaging in two different FOVs of chicken breast, despite their difference in absolute intensity integrated over one frame; (Bottom) observed 2PAF and 3PAF growth rates under pSLAM baseline/unchirped illumination (upper left) and chirped illumination (upper right), along with calculated rates of the latter according to 2nd order phototoxicity (lower left) and 1st order or linear phototoxicity (lower right). b) (Top) Illustration of spatial bin‐3 illumination in comparison to baseline/bin‐1 illumination; (Bottom) comparison of 2PAF and 3PAF growth rates according to bin‐1 (left) and bin‐3 pSLAM illumination (right), with Welch's *t*‐test showing significant statistical difference between the two (*p* < 0.0001). c) (Top) High WHF‐revealed phototoxicity but late cavitation (arrowhead) at a high pSLAM irradiance; (Middle) power‐dependent 3PAF growth rates under chirped and unchirped/baseline pSLAM illuminations that reveal nonlinear phototoxicity; (Bottom) lower WHF‐revealed phototoxicity but early cavitation (arrowhead) at a higher pSLAM power. d) (Top) Tri‐period eSLAM imaging of chicken breast that reveals linear phototoxicity at a low irradiance; (Bottom) Similar tri‐period imaging that reveals the emergence of nonlinear (photoionization‐accelerated) phototoxicity and existence of heating‐accelerated phototoxicity at a slightly higher irradiance. e) FLIM imaging of a chicken breast sample containing eSLAM imaging‐induced fluorescent compounds at a high irradiance, with color bar corresponding to lifetime in ns. Scale bar: 50 µm. f) (Left) Illustration of spatiotemporal bin‐3 illumination in comparison to baseline/bin‐1 illumination; (Right) comparison of 2PAF/3PAF growth rate per frame/pulse and SHG/THG intensity per pulse under bin‐1/3/10 pSLAM illumination. g) Determination of water optical breakdown threshold from the emergence of a 3PAF‐visible bubble (arrowhead) by pSLAM baseline “imaging” of 10 mm NADH solution. Scale bar: 50 µm. h) A simplified view of phototoxicity and related thresholds versus power/irradiance free of cumulative multipulse effect.

To evaluate the effect of pulse width^[^ ^8^, ^26^ ^]^ on 2PAF/3PAF linear growth rates (arbitrary unit per pulse), we compared two illuminations 10% above the corresponding WHF power thresholds (Table S6, baseline vs chirped, Supporting Information), which produced consistent data across different testing FOVs (**Figure** **3**a, top; and Figure S2, Supporting Information). The variation of observed growth rates among these FOVs may be attributed to subtle structural‐chemical variation because chicken breast is not a perfectly homogeneous sample (Figure S3, Supporting Information). By taking account of the dual role of excitation pulses to induce and detect WHF, the observed growth rates associated with the chirped illumination (Figure S4, left, Supporting Information) can be predicted from those associated with the baseline illumination according to an assumed phototoxicity photon order of either 1 or 2. Because the former is in quantitative agreement with experimental data (Figure 3a, bottom), the observed WHF is induced by linear absorption.

This result is surprising because muscle samples, such as chicken breast are not known as pigmented tissue, in contrast to mouse retina wherein the observed “luminescent flash” can be attributed to linear absorption/phototoxicity.^[^ ^31^ ^]^ It should be noted that two‐photon “photo‐enhancement” of rabbit red blood cells occurred more readily with longer pulses, implying a photon order of less than 2 (Table S4, Supporting Information). Moreover, the observed linear phototoxicity via WHF assay echoes that observed from mouse brain slices via the calcium microdomain hyperactivity in cortical astrocytes,^[^ ^32^ ^]^ which was attributed to the heating toxicity that can be detected offline after in vivo mouse brain imaging.^[^ ^33^ ^]^ These closely related results from different unlabeled samples strongly suggest that linear absorption of ubiquitous intrinsic NIR photosensitizers induce phototoxicity (Figure 1d).

### 2.3 Hyper‐Fluorescence Originates from Unrelaxed Triplet Rather Than Heating

To assess the plausible role of heating in WHF, we lowered the line rate of fast scanning direction (*x*) in the baseline illumination at one pulse per pixel (diffraction‐limited resolution of ≈0.4 µm) to bin 3 pulses spatially (Table S6, spatial bin, Supporting Information). Thus, one frame of the resulting bin‐3 illumination (3 pulses/pixel) took the same time and light dose as three frames of the baseline/bin‐1 illumination (1 pulse/pixel) (Figure 3b, top; and Figure S4, right, Supporting Information). The constant laser repetition rate (0.83 MHz) separated successive pulses apart by 1.2 µs, a period much longer than the thermal relaxation time of water (0.06–0.14 µs).^[^ ^7^, ^34^ ^]^ Thus, any heating effect in these illuminations would be dominated by single‐pulse heating with no interference between successive pulses, which would form the starting point to reveal plausible cumulative multipulse heating by increasing the repetition rate.^[^ ^7^ ^]^ In other words, WHF growth rates of the bin‐3 illumination would approximate those of the baseline illumination.

Surprisingly, the phototoxicity of 2PAF (or 3PAF) growth rate in the baseline illumination is only 21% (or 29%) of that in the bin‐3 illumination (Figure 3b, bottom; and Figure S4, right, Supporting Information), indicating a nonheating cumulative multipulse effect in the latter. As a result, the WHF power threshold in the bin‐3 illumination is systematically lower than that of the baseline illumination. It is thus unlikely that the observed WHF originates from direct heating.^[^ ^33^, ^35^ ^]^ In the context of reduced phototoxicity at an increased scanning speed,^[^ ^17^, ^18^, ^19^ ^]^ the simplest alternative interpretation of this effect is the unrelaxed triplet after the linear absorption of specific intrinsic NIR photosensitizers, with a µs‐scale lifetime prone to the excitation state absorption of successive pulses and subsequent high‐order triplet‐mediated phototoxicity^[^ ^16^ ^]^ (Figure 1d). In contrast to the bin‐3 illumination, the baseline illumination at 1 pulse/pixel avoids this phototoxicity by a faster scanning that spatially separates the individual pixels (excited photosensitizers in one cycle apart by ≈1 diffraction‐limited resolution) without the cumulative multipulse effect (Figure 3b, top), and thus relaxes the first‐order triplet (Figure 1d). This interpretation also validates the widely observed reduction in phototoxicity with increased fast‐axis scanning speed (Table S2, Supporting Information). Without laser scanning in a laser tweezer largely free of temperature rise and heating,^[^ ^36^ ^]^ the prolonged exposure (≈10 min) of to a trapped cell to cw‐NIR excitation with unrelaxed triplet^[^ ^16^ ^]^ would induce hyper‐fluorescence^[^ ^37^ ^]^ and related phototoxicity.^[^ ^20^ ^]^

### 2.4 Photoionization Accelerates Hyper‐Fluorescence at High Irradiance

To evaluate the power‐dependence of WHF growth rates, we employed the baseline and chirped bin‐1 pSLAM illuminations (largely free of the cumulative multipulse effect), except for larger powers up to ≈2‐time of the corresponding WHF power thresholds. Interestingly, both illuminations led to a nonlinear WHF phototoxicity with an apparent photon order within 3–6 (Figure S5, Supporting Information).

To identify the factor that accelerates the otherwise linear phototoxicity of WHF toward the nonlinear phototoxicity, we examine the dynamics of high phototoxicity well above the WHF power threshold (Figure 3c, middle). At the highest power of the baseline illumination, cavitation formed near the 80th frame (Figure 3c, top), whereas lower power did not cause cavitation after 120 frames (Figure S6, left, Supporting Information). In contrast, cavitation occurred at an earlier (≈15th) frame at the highest power of the chirped illumination, but the growth rate from either 3PAF or 2PAF was at least 50% lower (Figure 3c, bottom; and Figure S6, right, Supporting Information). Given a common temperature threshold for cavitation (110–130 °C),^[^ ^7^, ^10^ ^]^ the latter should sustain a higher thermal load than the former throughout the imaging. If the high‐irradiance heating dominates, a larger “WHF”‐revealed phototoxicity should be observed at a higher thermal load. However, the observed growth rate of “WHF” from 60‐fs 3.0 mW irradiation before a late cavitation (lower thermal load toward ≈130 °C) triples that from 300‐fs 5.9 mW irradiation before an early cavitation (higher thermal load toward ≈130 °C) (Figure 3c). Thus, WHF is mainly accelerated by single‐pulse photoionization dictated by irradiance (unit: TW cm^−2^), not the heating dictated by average power (unit: mW) or pulse energy (unit: nJ). The resulting photoionization‐accelerated WHF might overwhelm the otherwise progressively decreased SHG signal (Figure 3c, bottom) by thermal denaturing^[^ ^30^ ^]^ (arrow in Figure 3c, top).

### 2.5 Heating Accelerates Hyper‐Fluorescence at Low Irradiance

To generalize the results from pSLAM, we employed the eSLAM microscope that replaced the prism‐based compressor with a pulse shaper to electronically chirp 1110 nm pulses from 300 to 60 fs (Table S6, Supporting Information).^[^ ^29^ ^]^ With the same spatially separated pulsed excitation (i.e., bin‐1 illumination of the same FOV), the observed WHF power threshold increased from 1.8 mW in pSLAM to 17 mW in eSLAM (Figure S1, Supporting Information). However, the corresponding pulse energy (i.e., 2.2 nJ at 1030 nm in pSLAM vs 3.4 nJ at 1110 nm in eSLAM) are comparable, indicating the single‐pulse nature of phototoxic WHF (free of cumulative multipulse effect). This single‐pulse effect also dominates the cumulative multipulse effect in cavitation or heating toxicity (Figure S6, right, Supporting Information).

Using power 10% above the WHF power threshold (Table S6, eSLAM, Supporting Information), we conducted a tri‐period imaging experiment with switchable pulse chirping between successive periods. We observed the same linear WHF growth rate in the first and third periods that linked the 3PAF/2PAF‐revealed WHF by broken straight lines (Figure 3d, top). Thus, the same linear WHF growth was sustained by the second period, i.e., equivalent phototoxicity was attained by unchirped 60 fs pulses and chirped 300 fs pulses. However, due to the dual role of excitation pulses to induce and detect WHF, the phototoxicity in the second period could not be detected by chirped 300 fs pulses at a much lower signal‐to‐noise ratio (colored arrows; Figure 3d, top) in comparison to that from unchirped 60 fs pulses. This observation in eSLAM imaging independently confirms the linear absorption nature of WHF observed from pSLAM imaging (Figure 3a). More importantly, it highlights a unique advantage of short 60 fs pulses over longer pulses to sensitively detect phototoxicity via intrinsic WHF indicator.^[^ ^24^ ^]^

To examine the plausible role of heating, we used a power 20% above the WHF power threshold and observed a much larger WHF growth rate from the first period than that from the second period, which indicated the emergence of photoionization‐based acceleration (Figure 3d, bottom). Interestingly, the WHF growth rate from the third period surpassed that from the first period considerably (Figure 3d, bottom). If linearly absorbed light energy end ups more as heat than the photoionization‐accelerated WHF during the second period in comparison to the first period, we attribute the higher WHF growth rate of the third period than that of the first period to a higher global temperature.^[^ ^33^ ^]^ This implies that the global heating, which may be superimposed on the single‐pulse heating in eSLAM (with 0.2 µs pulse separation larger than the thermal relaxation time of 0.06–0.14 µs), accelerates WHF under a relatively low irradiance (Figure 1d). Similar acceleration of phototoxicity by heating has been reported in NIR phototherapy.^[^ ^23^ ^]^ As an implication, it allows detection of fundamentally linear phototoxicity by a chirping‐pulse experiment (Figure 3a, bottom and Figure 3d, top) but not a conventional power‐dependence experiment that would result in phenomenologically nonlinear phototoxicity (Figure 3c, middle; and Figure S5, Supporting Information).

### 2.6 Hyper‐Fluorescence Lifetime and Spectrum Generalizes Model Applicability

To further characterize the WHF‐emitting species, we invoked the built‐in FLIM capability of eSLAM based on computational photon counting.^[^ ^28^ ^]^ The WHF was first induced in one FOV (≈15 µm imaging depth) by 200 scans at a power 50% higher than WHF threshold to overwhelm its autofluorescence background, and then FLIM imaging was conducted on the same FOV and imaging depth at a power 10% below the WHF threshold. The lifetime of observed homogeneous WHF (≈1.5 ns) is longer than that of heterogenous WHF (≈0.6 ns; Figure 3e, arrowheads) formed later during the imaging (see Video S3, Supporting Information), both of which are comparable with those (0.5–1.6 ns) observed from cultured hamster kidney cells and ex vivo mouse kidney (Figure 2a,b; and Figure S7a–7c, Supporting Information). Similar lifetimes (≈1 ns) were obtained from the WHF of Chinese hamster cells^[^ ^38^ ^]^ and from the new fluorescent compounds in NIR‐ablated muscle and albumin samples,^[^ ^35^ ^]^ suggesting that similar fluorescent compounds produce both heterogeneous and homogeneous WHF in diverse cell/tissue samples (Table S5, Supporting Information).

The homogeneous WHF was also observed in a rat brain slice via 3PAF but not 2PAF across the FOV, whereas the latter could reveal this WHF in a central region of FOV with the highest irradiance (Figure S7d and Video S5, Supporting Information). From this result, we believe the photobleaching of strong autofluorescence background often obscures the homogeneous WHF, which would otherwise be widely observable in live samples. The intrinsically weak 2PAF/3PAF signals of chicken breast offer a rather clean background at the beginning of time‐lapse imaging for sensitive detection of the homogeneous WHF and accurate quantification of the phototoxicity.

To further characterize the red edge of observed WHF, we introduced a fifth detection channel of two‐photon excited long‐wavelength autofluorescence (2PLAF) into the eSLAM detection system with a detection band of 660–740 nm. At a power 50% higher than WHF threshold, we observed significant WHF signal from this channel in addition to 3PAF and 2PAF to cover a broad visible regime of 420–740 nm (Figure S8, Supporting Information). This result confirms the broad bandwidth of WHF from chicken breast and reinforces the notion of “white flashes” observed from Chinese hamster cells.^[^ ^38^ ^]^

### 2.7 Cumulative Multipulse Effect Lowers Imaging Performance

In contrast to the spatial bin‐3 illumination that binned 3 pulses into one spot spatially (Figure 3b, top), the more popular binning affordable by the flexible galvo–galvo scanning and tunable repetition‐rate laser of pSLAM was to bin multiple pulses into one spot spatiotemporally at the same fast‐axis scanning speed of the baseline illumination^[^ ^27^ ^]^ (Figures 2c,d and 3f, left). To evaluate the benefit/cost of this spatiotemporal binning, we performed imaging on different FOVs at bin‐1 (baseline), bin‐3, and bin‐10 illumination conditions with different average powers or repetition rates but the same peak power or pulse energy (Table S6, Supporting Information). Unlike the spatial binning case (Figure 3b, bottom), the WHF growth rate per pulse decreases with the bin number (Figure 3f, upper right). Although more detailed studies are needed to understand the observed decrease, this benefit is countered by the cost of lowered nonlinear signals including those from parametric SHG/THG processes (Figure 3f, lower right), which leave the quantum state of the material unchanged and do not involve triplet states. Thus, the observed signal reduction may be attributed to unrelaxed photoionization with up to 300 ns lifetime of solvated electrons and resulting large‐scale structural modulation via low‐density plasma.^[^ ^10^ ^]^ The corresponding cumulative multipulse effect produces similar nonlinear phototoxicity (Figure S5, Supporting Information) but lowers the WHF irradiance threshold due to a higher WHF growth rate per frame (Figure 3f, upper right). Thus, it is more beneficial to increase the throughput of the pSLAM baseline imaging by the bin‐1 (no‐bin) illumination of eSLAM, free of the cumulative multipulse effect that may impair embryo development.^[^ ^39^ ^]^ This eSLAM illumination relaxes the linear‐absorption‐mediated triplet (Figure 1b) by combining a higher repetition‐rate excitation with proportionally faster scanning and thus attains the same bin‐1 illumination in pSLAM (Table S6, Supporting Information).

To estimate the optical breakdown threshold of water defined by the bubble formation,^[^ ^10^ ^]^ which is a single‐pulse effect different from the cumulative pulse‐induced cavitation, we conducted the pSLAM baseline illumination on a 10 mm solution of reduced nicotinamide adenine dinucleotide (NADH) at different pulse energies and identified a threshold of 5.3 nJ (Figure 3g) corresponding to 9.3 TW cm^−2^ at 1030 nm (similar threshold of ≈9 TW cm^−2^ was also attained at 1110 nm). This threshold approximates the theoretical value of 3.9 nJ or ≈7 TW cm^−2^ (1064 nm, 100 fs, NA 1.3).^[^ ^10^ ^]^ Thus, gentle imaging free of unrelaxed triplet (detectable WHF in Figure S1, Supporting Information) can be conducted at a surprisingly large ratio (≈50%) of water breakdown threshold (normalized irradiance in Table S7, Supporting Information). The irradiance/power‐dependent phototoxicity becomes clear without the interfering cumulative multipulse effect (Figure 3h).

### 2.8 Triplet‐Relaxation Enables High‐Performance Imaging

**Figure 4:**
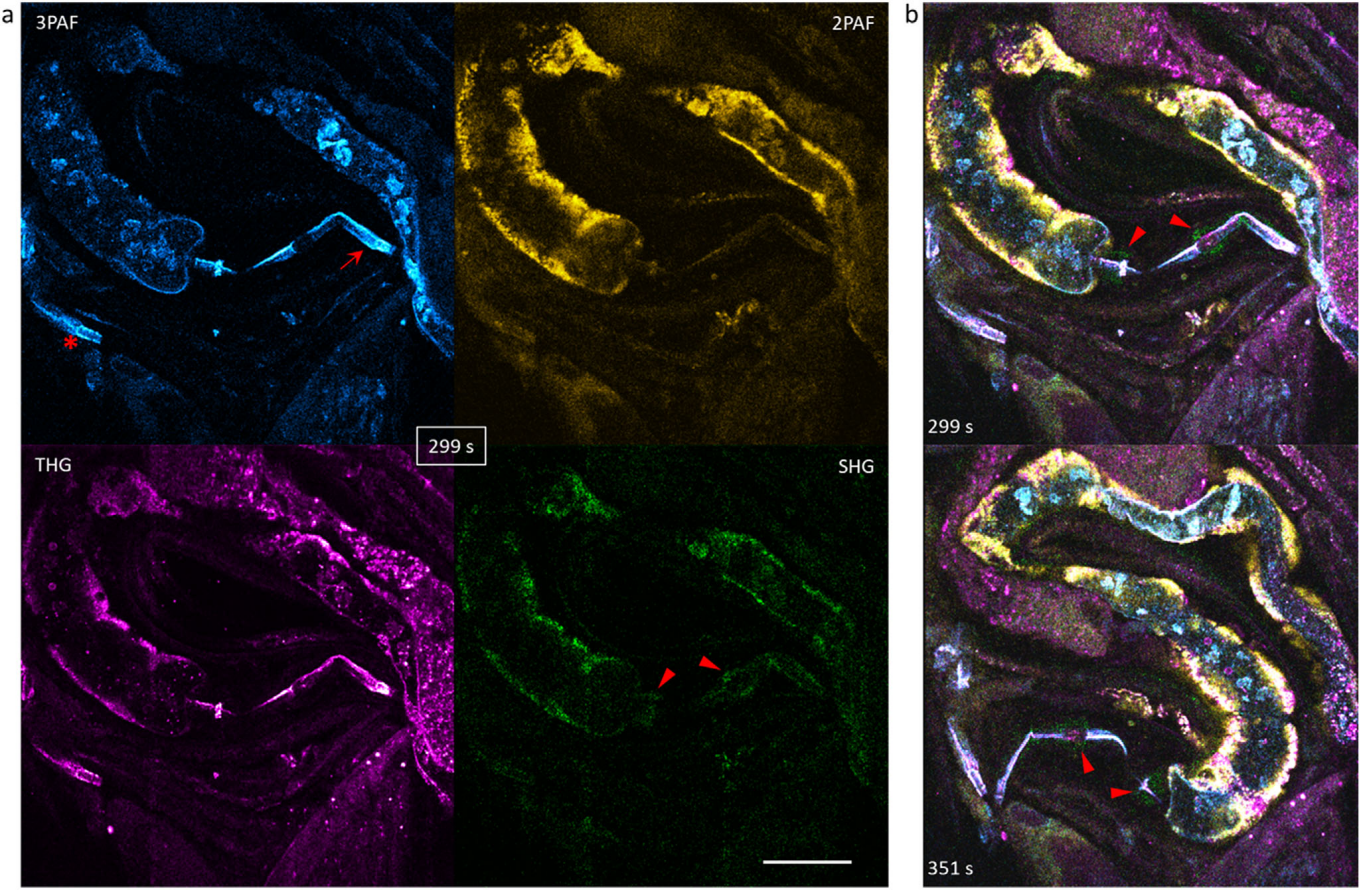
Gentle eSLAM imaging of unlabeled moving *C. elegans* (14.4 mW on sample). a) Instant single‐frame images of one worm with 3PAF/cyan‐visible pharynx (arrow) and a pair of SHG/green‐visible bulbs (arrowheads); similar pharynx structure (star) indicate the presence of multiple worms in the same field of view. b) Related time‐lapse imaging of the worm (arrowheads) with 0.33 s exposure per frame and 1.37 s between successive frames; related video reveals THG/magenta‐visible embryos and uterus, 3PAF/cyan‐visible proximal and distal gonads with internal germ cells or oocytes, 3PAF/THG‐visible intestine, and 2PAF/yellow‐visible body wall muscle (Video S6, Supporting Information). Scale bar: 50 µm.

Just like in linear optical imaging,^[^ ^17^, ^18^, ^19^ ^]^ the triplet states can be relaxed by increasing the fast‐axis scanning speed to decrease the pulses per diffraction‐limited‐resolution (PPD) (Table S2, Supporting Information). The PPD parameter is equivalent to the variable bin number in pSLAM and can be lowered to ≈1 like in eSLAM. To highlight the high‐performance eSLAM imaging, we investigated wide‐type unlabeled *C. elegans* in standard culture (OP50). All signals were epi‐detected and displayed at 0.73 Hz frame rate (1024×1024‐pixel frame with a FOV of 250×250 µm^2^) without image denoising/reconstruction. We observed the free motion of one worm with 0.2 µs pixel dwell time and identified its moving 3PAF‐visible pharynx and related pair of SHG‐visible bulbs (**Figure** **4**, arrowheads). It is informative to compare our eSLAM imaging with a conventional imaging method of *C. elegans* (excitation: 750 nm, 240 fs, 80 MHz, 15 mW) with comparable acquisition parameters (FOV: 180×180 µm^2^; frame: 512×512 pixels; 2.5 µs pixel dwell time; frame rate: 0.15 Hz).^[^ ^28^ ^]^ The eSLAM imaging (Video S6, 409.6 s, Supporting Information) provides more information content at a higher signal‐to‐noise ratio and a faster imaging speed in comparison to the video (Video S2, 135.7 s, Supporting Information) in that study.

The “gentle” but high‐performance eSLAM imaging originates from the triplet‐relaxation that avoids the cumulative multiple‐pulse effect via a high fast‐axis scanning speed. This strategy can be conceptually divided into two independent steps. The first step is to maximize single‐pulse signal generation, i.e., *E^n^/τ^n^* ^−1^ for *n*‐photon excitation processes^[^ ^40^ ^]^ (where *E*, *τ*, and *f* are the energy, temporal width, and repetition rate of excitation pulse at a fixed central wavelength), by increasing *E* and/or decreasing*τ* until a phototoxicity threshold free of cumulative multiple‐pulse effect (Figure 3a). By attaining PPD ≈ 1 with minimum single‐pulse photoionization, gentle pSLAM imaging can be conducted at >40% ratio of water optical breakdown, much higher than the ratio of 8.6% that enables laser surgery and the ratio of <3% that enables imaging‐induced phototoxicity (Table S7, Supporting Information). The second step is to increase signal generation rate by increasing *f* and fast‐axis scanning speed proportionally (PPD ≈ 1) until a phototoxicity threshold of global heating (Figure 3d, bottom), paralleling the shift from pSLAM to eSLAM imaging (from *f* = 0.83 MHz to *f* = 5 MHz). Thus, the combination of the two steps should enhance the phototoxicity‐free signal generation rate that scales with (*E^n^/τ^n^* ^−1^)*f*.

For example, in comparison with a well‐known gentle condition to image mammalian embryos^[^ ^41^ ^]^ (Table S2, Supporting Information) at 1047 nm excitation, 13 mW on sample, and ≈690 PPD (*E* = 0.108 nJ, *τ* = 175 fs, *f* = 120 MHz), our eSLAM imaging at comparable excitation wavelength (1110 nm) and power (14.4 mW) but a lower PPD of ≈1.5 (*E* = 2.88 nJ, *τ* = 60 fs, *f* = 5 MHz) should enhance the phototoxicity‐free signal generation rate by a factor of 86 for two‐photon excitation processes. This enhancement may have enabled our detection of the SHG‐visible bulbs in a reflective configuration (Figure 4, arrowheads). It is unprecedented to observe strong back‐reflected SHG signals from transparent samples such as *C. elegans*, as the SHG‐visible bulbs have only been detected using less adaptive transmission configuration with 5 µs pixel dwell time that necessitated animal immobilization.^[^ ^42^ ^]^ In contrast to the enhancement factor of 86 for two‐photon excitation processes, this factor becomes 6.7×10^3^ (or 5.2×10^5^) for three‐photon (or four‐photon) excitation processes. Indeed, preliminary experiments have demonstrated the gentle eSLAM imaging of cellular tryptophan by four‐photon‐excited autofluorescence.^[^ ^43^ ^]^

## 3 Discussion

One major confusion regarding pulsed NIR phytotoxicity (Table S1, Supporting Information) arises from different relaxation times of >1 µs (photochemistry), ≈0.3 µs (photoionization), and ≈0.1 µs (heating) that produce different extents of cumulative multipulse effect (Figure 3b,f). This difference is prevalent in the typical illumination (galvo–galvo scanning of ≈80 MHz pulses) of nonlinear optical imaging (Table S2, Supporting Information) and may have obscured the mechanism hypothesized in this study. By avoiding this cumulative multipulse effect, we attribute the phototoxicity more to unrelaxed triplet and subsequent heating‐induced acceleration (Figure 1d) than direct heating.^[^ ^33^, ^35^ ^]^ Interestingly, cumulative multipulse heating can accelerate the fundamentally linear phototoxicity^[^ ^32^ ^]^ to resemble the phenomenologically nonlinear phototoxicity with prominent triplet relaxation (PPD ≈ 6) observed in the picosecond excitation of coherent anti‐Stokes Raman scattering microscopy^[^ ^44^ ^]^ (Table S7, Supporting Information). This pseudosingle‐pulse effect is further supported in the form of single‐pulse heating (Table S1, Supporting Information) by the low phototoxicity onset below 10% of water optical breakdown in pigmented specimens of skin^[^ ^7^ ^]^ and retina^[^ ^31^ ^]^ (Table S7, Supporting Information), and the absence of reported WHF in UV–visible confocal fluorescence microscopy.

Another confusion arises from the coexisting linear and nonlinear NIR phototoxicity at different thresholds (Figure 3h). The observation of this coexistence in our WHF assay (at low and high powers, respectively) echoes that in a previous study at ≈30‐fold lower irradiance^[^ ^45^ ^]^ (Table S7, Supporting Information) and reconciles the existence of either linear or nonlinear NIR phototoxicity in various WHF‐like assays (Table S4, Supporting Information) and bioassays (Table S8, Supporting Information). Phenomenological nonlinear phototoxicity originates from the linear NIR absorption^[^ ^32^, ^45^ ^]^ of ubiquitous intrinsic NIR photosensitizers,^[^ ^20^, ^21^, ^22^, ^23^ ^]^ just like that from the excessive (high‐power) linear absorption of intrinsic UV–visible photosensitizers.^[^ ^32^ ^]^ Thus, the apparent photon order of ≥2 in the observed phototoxicity (Tables S4 and S8, Supporting Information) does not imply a multiphoton absorption, as would be commonly assumed (Figure 1b). In other words, the concept of photon order (>1) is useful to characterize the nonlinearity of molecular absorption and harmonic generation but not a phenomenological nonlinear phototoxicity (that may be of linear origin). Without this “evidence” that has strongly supported the nonlinear‐absorption‐mediated phototoxicity (Figure 1b), all other evidence can be reinterpreted to be compatible with our hypothesized mechanism of linear‐absorption‐mediated (fundamentally linear) but phenomenologically nonlinear phototoxicity (Figure 3h; and Table S9, Supporting Information), and to reconcile with the observed linear‐absorption‐mediated phototoxicity in nonpigmented samples (Tables S4 and S8, Supporting Information).

With the recognition of both confusions, mitigating phototoxicity in clinically relevant label‐free (in vivo) nonlinear optical imaging (Table S3, Supporting Information) resembles that of photobleaching under fluorescently labeled imaging,^[^ ^16^ ^]^ i.e., triplet relaxation. Like the fluorescently labeled imaging using rapidly scanned cw excitation,^[^ ^17^ ^]^ the triplet relaxation in eSLAM is attained by rapid resonant‐galvo scanning of 5 MHz pulses to avoid the cumulative multiple‐pulse effect, so that subsequent pulses address well‐resolved pixels ≈1 diffraction‐limit resolution apart (i.e., PPD ≈ 1). Neutralizing heat generation may further mitigate phototoxicity in vitro.^[^ ^20^, ^23^ ^]^ In this way, the unfulfilled potential of laser‐scanning nonlinear optical microscopy^[^ ^6^ ^]^ in gentle imaging^[^ ^3^ ^]^ may be recovered by a surprisingly simple strategy widely accessible by microscopists.

The proposed strategy is not intrinsically incompatible with the roadmap on label‐free super‐resolution imaging^[^ ^46^ ^]^ and popular FLIM based on time‐correlated single photon counting (TCSPC) over a rather long pixel dwell time.^[^ ^47^ ^]^ We note that a more complete triplet‐relaxation by “sub‐bin‐1” illumination can be achieved by using superfast <1 pulse/pixel scanning.^[^ ^48^ ^]^ It is thus possible to attain gentle super‐resolution eSLAM imaging by combining this unique illumination (PPD < 1) with a reported deconvolution method.^[^ ^49^ ^]^ As to FLIM, we have replaced TCSPC with high‐speed temporal sampling^[^ ^28^ ^]^ to measure fluorescence lifetime (Figure S7, Supporting Information). Temporal sampling by a faster (e.g., 10 GS s^−1^) digitizer can improve the temporal resolution to approach that from TCSPC. Thus, our strategy to mitigate phototoxicity does not exclude super‐resolution and FLIM imaging, even though it appears as a compromise between the need to mitigate phototoxicity by triplet relaxation and a seemingly conflicting need to recover diffraction‐limited spatial resolution via Nyquist condition‐fulfilled sampling.

It is possible to link WHF phototoxicity from unrelaxed triplet states to the generation of reactive oxygen species (ROS).^[^ ^2^, ^3^ ^]^ Unfortunately, simultaneous use of our WHF assay with a ROS‐specific fluorescent assay^[^ ^25^ ^]^ cannot rule out the pre‐exposure perturbation to the physiological environment by the foreign materials of the ROS assay.^[^ ^50^ ^]^ An offline assay after exposure, reported in a recent study on cultured cells,^[^ ^51^ ^]^ requires a thin sample and would also perturb the physiological environment of intact 3D tissue by fresh frozen sectioning. To avoid any perturbations to the physiological environment, label‐free indirect detection of ROS has been explored but the method is limited to lipid‐rich cells.^[^ ^50^ ^]^ Thus, more work is needed to clarify the role of ROS in the observed WHF phototoxicity, and plausible role of free‐electron‐mediated pathways at levels well below the water breakdown threshold.^[^ ^10^ ^]^ Similarly, more detailed studies are needed to examine whether the widely decreased NIR phototoxicity with fast‐axis scanning speed across 700–1200 nm illumination (Table S2, Supporting Information) implies a negligible reverse inter‐system crossing for high order triplet states, which can mitigate the photobleaching of fluorescent proteins^[^ ^52^ ^]^ (Figure 1d).

Despite the uncertainties on ROS and reverse inter‐system crossing, a general guide to optimize pulse parameters for gentle imaging is possible. Specifically, short (sub‐80‐fs) pulse^[^ ^31^, ^53^ ^]^ is preferred over longer (picosecond) pulses^[^ ^44^, ^45^ ^]^ to not only increase nonlinear signal generation near phototoxicity onset^[^ ^32^ ^]^ but also detect this onset more sensitively than 300‐fs pulses (Figure 3d, top) within a narrow window of fundamentally linear phototoxicity (Figure 3h). On the other hand, the low end of pulse width will be largely limited to ≈30 fs by the chromatic dispersion and aberration of the microscope. As to the pulse repetition rate, the choice depends on the trade‐off between imaging depth and speed as well as the constraint by global heating.^[^ ^33^ ^]^ The optimal repetition rate may lie within the 5–20 MHz range but not near GHz range.^[^ ^54^ ^]^ Despite the focus on label‐free nonlinear optical imaging, it is reasonable to assume that these optimal pulse parameters and the general strategy of triplet relaxation are valid for more complicated imaging scenarios with extrinsic fluorescent labeling.

Even though label‐free WHF cannot replace existing (more subtle or long‐term) phototoxicity assays based on ROS or other biological functions of interest (Table S8, Supporting Information), it could complement the latter to optimize imaging condition due to its broad sample‐independent applicability. The intrinsic WHF phototoxicity indicator avoids the limitations of the photobleaching of specific extrinsic fluorophores^[^ ^2^, ^3^ ^]^ and the prohibition of plausible toxic phototoxicity assays in clinical imaging. The required high‐speed laser scanning for triplet relaxation allows real‐time sensitive monitoring of phototoxicity and unambiguous identification of a nonfluence (power or irradiance) threshold with minimal doses (from early frames in Figure S1, Supporting Information). Thus, an otherwise complicated fluence threshold can be replaced by a simpler power or irradiance threshold^[^ ^14^ ^]^ (Figure 3h) to detect phototoxicity in real‐time and thus restrict further damage to nucleus^[^ ^8^, ^24^ ^]^ and morphology^[^ ^38^, ^45^ ^]^ (Table S1, Supporting Information). Interestingly, the emergence of WHF in chicken breast reveals the otherwise invisible myofibrils^[^ ^55^ ^]^ (Figure 2d), suggesting the origin of fluorescent Schiff base in lipofuscin‐like products from the peroxidation of polysaturated lipids that crosslinks proteins.^[^ ^24^, ^25^ ^]^

One limitation of this study is the use of chicken breast, which is amendable for quantitative measurements and modeling due to a highly homogeneous microstructure, to broadly represent biological samples. It is likely that the phototoxicity from other biological samples with typically heterogeneous microstructures would originate from different photosensitizers and generate different WHF‐emitting compounds. However, two lines of evidence suggest chicken breast as a representative model to study phototoxicity. First, the WHF lifetimes from chicken breast and other samples (hamster kidney cells, mouse kidney tissue, and rat brain) are all around 1 ns (Figure S7, Supporting Information), implying similar chemicals have been generated to emit heterogeneous and homogeneous WHF (Table S5, Supporting Information). Second, we have tested the eSLAM imaging in diverse cultured cells and tissue specimens.^[^ ^43^ ^]^ Rather independent of sample types, a 10% higher irradiance than the obtained threshold from chicken breast consistently generates WHF in time‐lapse imaging whereas a 10% lower irradiance consistently avoids the WHF. This test also validates WHF as a general inline indicator for phototoxcity.^[^ ^24^ ^]^ Another limitation of this study is the narrow range of central excitation wavelengths (1030–1110 nm) due to available femtosecond laser sources. The dependence of phototoxicity on excitation wavelength may rely more on the absorption properties of photosensitizers (initiator) and plausible heat‐generating pigments or chromophores (accessory) than those of the resulting species emitting WHF (end‐product) (Figure 1d). More studies on diverse biological samples and their overall action spectrum of NIR phototoxicity^[^ ^20^, ^21^ ^]^ will be needed to determine whether there exists an optimal excitation wavelength within 700–1300 nm for gentle nonlinear optical imaging. For label‐free in vivo clinical imaging, it will be interesting to examine whether 3PAF imaging of NADH in pigmented samples at 1110 nm (eSLAM) could outperform conventional 2PAF imaging of cellular NADH at 750–780 nm^[^ ^7^, ^31^ ^]^ due to low linear absorption of melanin at longer wavelengths.^[^ ^56^ ^]^ For basic science, it will be important to study wavelength‐dependent triplet generation and relaxation at typical illumination conditions.

## 4 Experimental Section

### Cell Culture

Adherent Syrian golden hamster kidney fibroblast cells (BHK‐21, clone 13, ATCC #CCL‐10) were cultured in disposable BioLite 75 cm^2^ vented‐cap cell culture treated flasks according to supplier‐recommended protocols. They were maintained inside a humidified incubator with 5% CO_2_ and 21% O_2_ conditions at 37 °C. A 0.5–1 mL volume of harvested cells was resuspended in 1.5–1 mL of phenol red‐free GibcoTM 1X TrypLE Select Enzyme (pH 7.0–7.4) cell dissociation reagent (TFS, Cat #12563029) in triplicates. The cells were imaged within 10 min of the resuspension.

### C. elegans

*C. elegans* growing on agar plates seeded with *E. coli* were obtained from Carolina Biological Supply Company. After additional growth of 2–4 days, a small portion was cut out of the agar plate and placed in a dish (P35G‐0‐10‐C, MatTek) for imaging.

### Vertebrate Animals

Fresh chicken breast was purchased from a local supermarket, cut by a razorblade with a smooth surface, and imaged within 24 h. All experiments on rodents were performed in compliance with the Guide for Care and Use of Laboratory Animals of the National Institutes of Health and approved by the Institutional Animal Care and Use Committee at the University of Illinois at Urbana‐Champaign (Animal Welfare Assurance No. A3118‐01). Brains of 4‐week Long‐Evans rats from an inbred colony (LE/BluGill) were removed and immersed in ice‐cold slicing media (93 mm *N*‐Methyl‐D‐glucamine, 2.5 mm KCl, 1.2 mm NaH2PO4, 30 mm NaHCO3, 20 mm HEPES, 25 mm glucose, 2 mm Thiourea, 3 mm Sodium pyruvate, 10 mm MgSO4, 0.5 mm CalCl2, pH 7.4) bubbled with CO_2_. Coronal slices (400 µm) containing the medial suprachiasmatic nucleus (SCN) were sectioned by a vibratome (Leica VT1000S). Slices were transferred to tissue culture inserts (0.4 µm; Millicell‐CM, Millipore) contained within 35 mm tissue culture dishes. The dishes were immersed in 1 mL of organotypic media, i.e., DMEM without sodium pyruvate supplemented with 10 mm HEPES, GS21 (1:50, GlobalStem), Penicillin‐Streptomycin (1:100, ThermoFisher Scientific) and 1 mm L‐glutamine. Cultures were kept at 37 °C in 5% CO_2_ and media were exchanged every other day. Brain slices kept in culture for <1 week were used for imaging. Mice (C57BL/6J, Jackson Laboratory) were used to obtain ex vivo kidney samples and fresh red blood cell samples, which were imaged directly without specific preparation.

### Imaging Instrumentation

A custom fiber supercontinuum laser^[^ ^57^ ^]^ with spectral broadening in a photonic crystal fiber was built based on a compact ultrafast fiber laser source (Satsuma, Amplitude Laser) emitting specific femtosecond pulses (1030±5 nm, ≈350 fs, tunable repetition rate 1–40 MHz, and up to 10 W). This custom fiber supercontinuum laser was used for either pSLAM (1030±40 nm, 60 fs, 0.83–20 MHz)^[^ ^27^ ^]^ or eSLAM imaging (1110±30 nm, 60 fs, 5 MHz).^[^ ^29^ ^]^ In this study, the pSLAM imaging offered more flexible excitation with a tunable pulse repetition rate (0.83–8.3 MHz) and a tunable scanning line rate (Figure 3a,b,f), while the eSLAM imaging supported a faster but fixed scanning line rate (1592 Hz) at a fixed pulse repetition rate of 5 MHz. The corresponding inverted pSLAM (or eSLAM) microscope employed raster laser scanning by a galvo–galvo (or resonant‐galvo) mirror pair under a high UV‐transmission objective (UAPON 40XW340, N.A. = 1.15, Olympus). Data acquisition software of the pSLAM (or eSLAM) microscope was developed based on ScanImage from MBF Bioscience (or LabVIEW from National Instruments). All imaging experiments were conducted at room temperature with no additional temperature control of cell/tissue samples. Main independent parameters of the two systems are compared in Table S6 (supporting Information). The reflective signals generated from the sample were spectrally separated into 4 detection channels of THG, 3PAF, SHG, and 2PAF by long‐pass dichroic mirrors and bandpass filters from Semrock, Inc., and subsequently detected by 4 analog photomultipliers from Hamamatsu. Common bandpass filters were used to detect 3PAF (417–477 nm) and 2PAF (593–643 nm), whereas different bandpass filters were used to detect THG (335–347 nm for pSLAM vs 365–375 nm for eSLAM) and SHG (498–520 nm for pSLAM versus 543–566 nm for eSLAM). The chirped 300 fs and unchirped 60 fs pulses at the focus of the microscope objective was enabled by either a prism‐based (pSLAM: BOA‐1050, Swamp Optics) or a spatial light modulator‐based pulse compressor (eSLAM: femtoJock Box, BioPhotonic Solutions Inc.), the latter of which allowed agile switch between the chirped and unchirped pulses (Figure 3d).

### Instrument Characterization

The M^2^ value of pSLAM laser source (1.10) or eSLAM laser source (1.16) was measured by a commercial device (M2MS, Thorlabs) to calculate irradiance. The pulsed width of collimated incident laser beam in pSLAM (or eSLAM) microscope was measured by a commercial autocorrelator (Mini TPA/PD, APE GmbH), while the shortest pulse width (i.e., unchirped 60 fs FWHM) at the focus of the microscope objective was determined by adjusting the pulse compressor to maximize the THG signal from a microscope coverslip under laser‐scanning illumination (i.e., imaging). The corresponding chirped 300 fs pulses were generated by introducing to the pulse compressor the known linear chirp from 60 fs pulse to 300 fs. The point spread function (PSF) of the eSLAM microscope was found to approximate the diffraction limit. The total transmission efficiency between the scanning mirror pair and sample was measured by one optical power meter after the scanning mirror pair (PMKIT‐21‐01, Newport) and another optical power meter after the microscope objective with a defocused spot of a few mm diameter (S175C, Thorlabs). The measured total transmission efficiency agreed with that of all intermediate optical elements (including microscope objective) from the corresponding vendors. The average power on sample was therefore determined by multiplying the power measured after the scanning mirror pair with the calibrated total transmission efficiency (35% for pSLAM and 21% for eSLAM) and was adjusted in the range of 0–25 mW by a neutral density filter wheel.

### Built‐In FLIM

Time‐resolve photon counts were determined using the reported computational photon counting.^[^ ^28^, ^29^ ^]^ Two analog‐output PMTs (H7422A‐40, Hamamatsu) were used to collect 2PAF and 3PAF signals, which were amplified by a high speed transimpedance amplifies (C5594, Hamamatsu) and digitized at 2 GS s^−1^ with a high‐speed digitizer (ATS 9373, AlazarTech). After acquisition, data was processed using custom Matlab (Mathworks) code to perform phasor analysis, estimating the mean lifetime and phasor components *g* and *s*. Phasor analysis was chosen in order to provide an unbiased lifetime estimation (unlike a two‐component bi‐exponential decay model) due to the previously unknown characteristics of WHF. Assisting by the videos (Videos S1, S2, and S5, supporting Information) to identify both the heterogeneous WHF and homogeneous WHF, the corresponding regions in the field‐of‐view were manually segmented for region‐specific analysis (Figure S7, supporting Information).

### Phototoxicity Modeling

Assuming the dual role of excitation pulse to induce and detect WHF in chicken breast via the linear/nonlinear optical processes of laser‐scanned illumination and imaging, the observed WHF growth rate at a constant pulse repetition rate is proportional to the product of WHF induction efficiency and WHF detection efficiency, i.e., (*P^m^*/*τ^m^* ^−1^)×(*P^n^*/*τ^n^* ^−1^), where *P* is average power, *τ* is pulse width, *m* is the photon order to induce WHF, and *n* is the photon order to detect WHF (*n* = 2 for 2PAF and *n* = 3 for 3PAF). Given the experimental WHF growth rates from unchirped pulses (*P* = 2.0 mW, *τ* = 60 fs) detected by 2PAF and 3PAF, the related theoretical WHF growth rates corresponding to the chirped pulses (*P* = 4.0 mW, *τ* = 300 fs) and an assumed photon order to induce WHF (*m* = 1 for 1st order or linear and *m* = 2 for 2nd order or nonlinear) can be calculated according to this product (Figure 3a, bottom). The theoretical WHF growth rates with an assumed photon order of 1 are in good agreement with the experimental WHF growth rates from the chirped pulses, indicating that the observed WHF is induced by a linear absorption process (Figure 3a, bottom).

## Conflict of Interest

The authors declare no conflict of interest.

## Author Contributions

G.W. and H.T. conceived the idea. H.T. and J.C. obtained funding for this research. L.L. and J.C. proposed and tested chicken breast model to study phototoxicity. G.W., J.E.S., and H.T. conducted related experiments. L.L., G.W., J.E.S., and H.T. performed data analysis and drafted the manuscript. H.T. and J.C. reviewed and edited the manuscript with inputs from all authors.

## Supporting information



Supporting Information

Supplemental Video 1

Supplemental Video 2

Supplemental Video 3

Supplemental Video 4

Supplemental Video 5

Supplemental Video 6

## Data Availability

The data that support the findings of this study are available in the supporting Information of this article.
