# Supplementary material for: Gentle Label‐Free Nonlinear Optical Imaging Relaxes Linear‐Absorption‐Mediated Triplet
Source: Adv Sci (Weinh). 2025 May 28;12(32):e15648. doi: 10.1002/advs.202415648 (PMC12407279; doi:10.1002/advs.202415648)
Supplement: Supplementary file 1 — Supporting Information [file ADVS-12-e15648-s005.docx]

Supporting Information

**Gentle label-free nonlinear optical imaging relaxes linear-absorption-mediated triplet**

Geng Wang^1,2^, Lianhuang Li^3*^, Janet E. Sorrells^2^, Jianxin Chen^3^*, Haohua Tu^1,2^*

^1^Department of Electrical and Computer Engineering, University of Illinois at Urbana-Champaign, Urbana, IL, 61801, USA

^2^Beckman Institute for Advanced Science and Technology, University of Illinois at Urbana-Champaign, Urbana, IL, 61801, USA

^3^Key Laboratory of OptoElectronic Science and Technology for Medicine of Ministry of Education, Fujian Provincial Key Laboratory of Photonics Technology, Fujian Normal University, Fuzhou, 350007, China

*Corresponding author. Email: lhli@fjnu.edu.cn, chenjianxin@fjnu.edu.cn, and htu@illinois.edu.

**
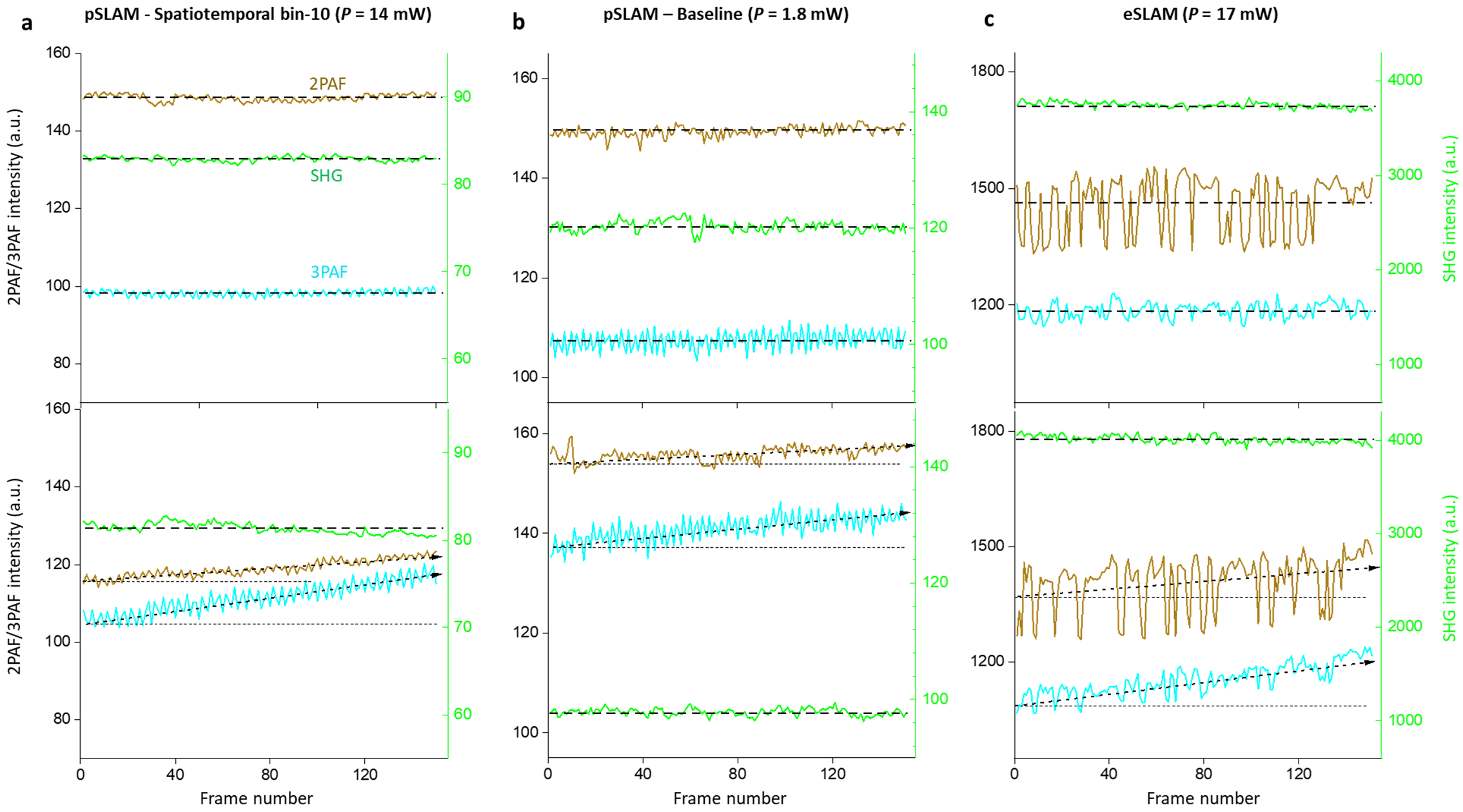
**

**Fig. S1** **Power threshold to generate WHF in chicken breast model by pSLAM (left two panels) or eSLAM (right panel)**. At the power threshold, spatially integrated 2PAF and 3PAF (i.e., WHF) signals may not (upper panel) or may (lower panel) increase with frame number during time-lapse imaging, using spatially integrated SHG signal as reference. Details of three illumination conditions other than power *P* are shown in Table S6.

**
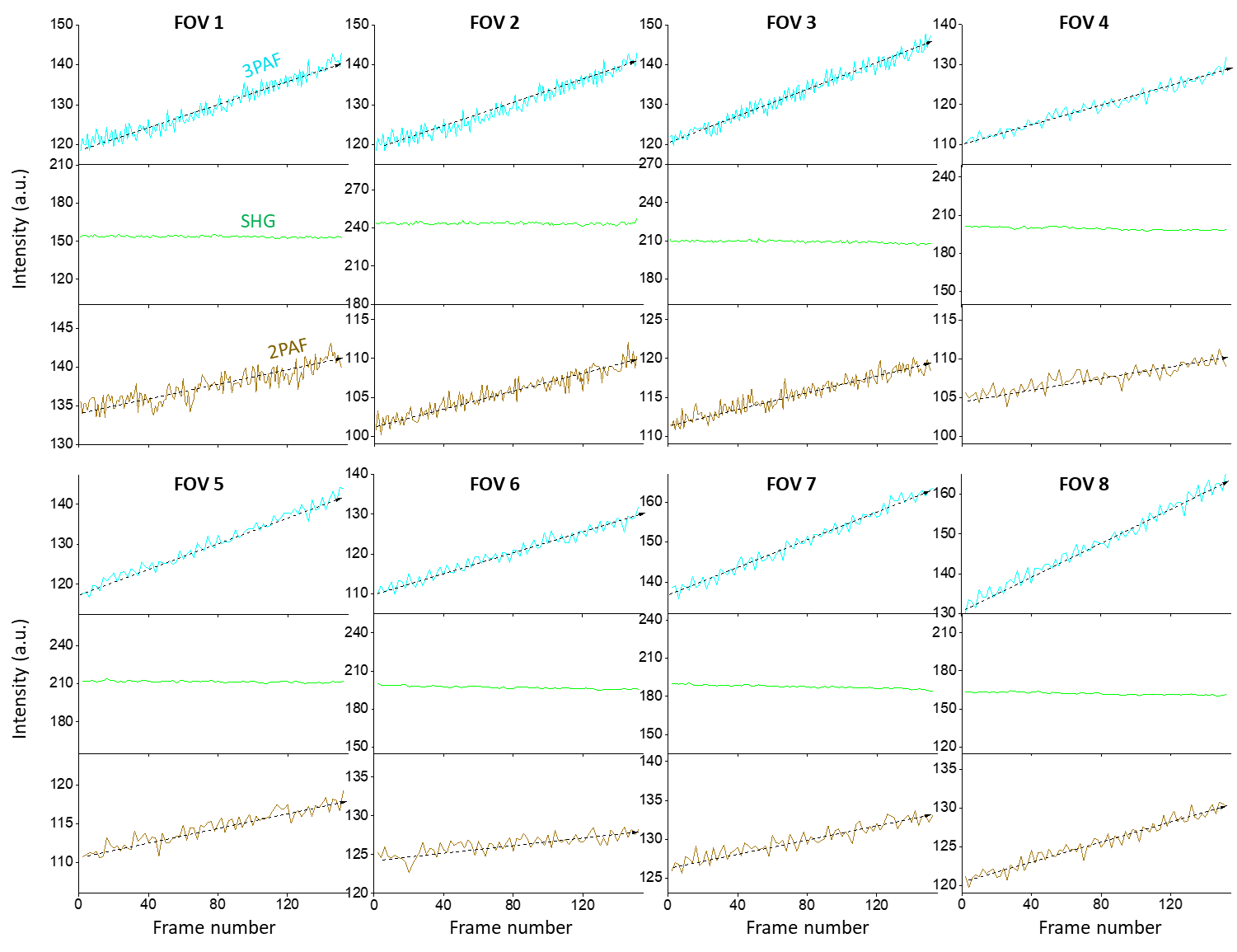
**

**Fig. S2** **Reproducible 3PAF and 2PAF growth rates under the baseline pSLAM illumination across different FOVs in chicken breast.** Spatially integrated 2PAF, 3PAF, and SHG signals during time-lapse imaging are plotted in the same intensity scales despite their difference in absolute intensity across FOVs.

**
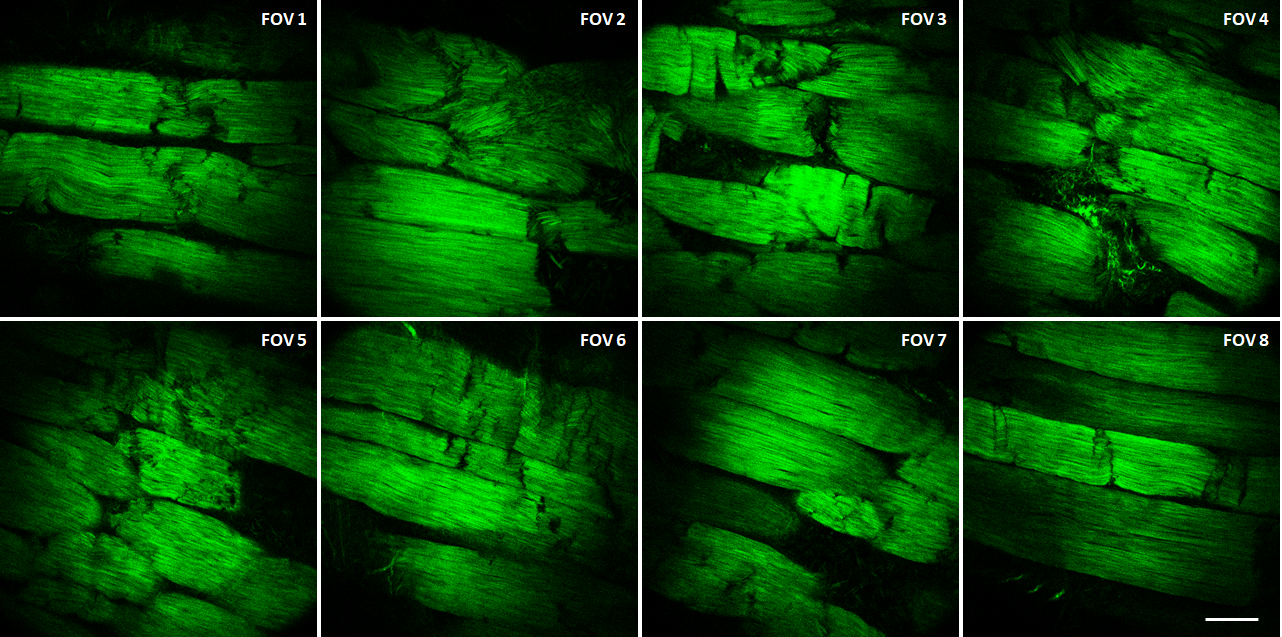
**

**Fig. S3 Subtle structural-chemical variation among different FOVs of chicken breast observed from SHG-sensitive myosin.** This variation may be responsible for the corresponding variation of WHF growth rates (see Fig. S2). Scale bar: 50 µm.

**
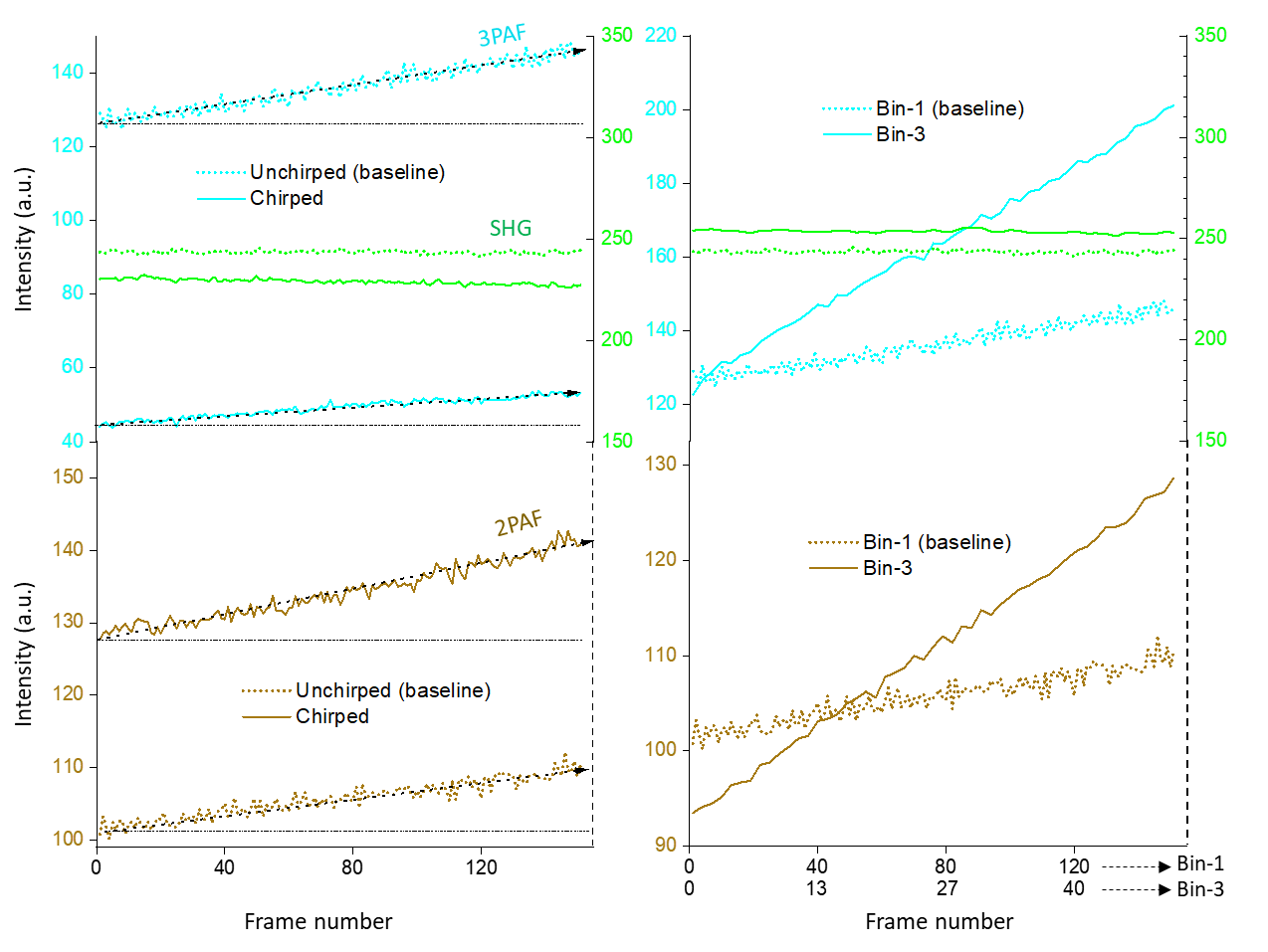
**

**Fig. S4** **Effect of pSLAM pulse chirping (left) or pulse spatial binning (right) on WHF growth rates.** In comparison to unchirped/baseline illumination (60 fs, 2.0 mW), chirped illumination (300 fs, 4.0 mW) attains a higher 2PAF growth rate but a lower 3PAF growth rate. In comparison to bin-1 illumination (baseline), bin-3 illumination (60 fs, 2.0 mW) attains much higher 2PAF and 3PAF growth rates (note that signal intensity of the bin-3 illumination integrated over one frame is normalized by the number of binning, i.e. 3).

**
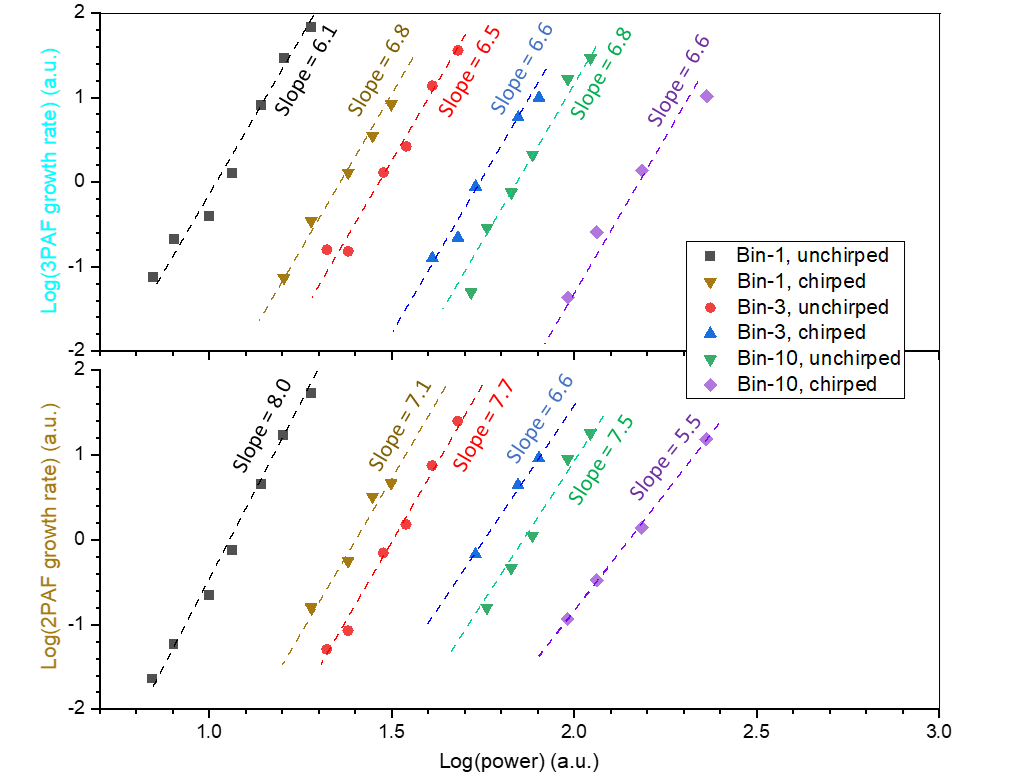
**

**Fig. S5** **Apparent photon orders of WHF growth rates revealed by 3PAF (top) and 2PAF (bottom) with/without pSLAM pulse chirping or spatiotemporal binning.** The apparent photon orders vary across 6.1-6.8 for 3PAF (photon order 3), indicating a phenomenological nonlinear phototoxicity of order 3.1-3.8. The related apparent photon orders vary across 5.5-8.0 for 2PAF (photon order 2), indicating a phenomenological nonlinear phototoxicity of order 3.5-6.

**
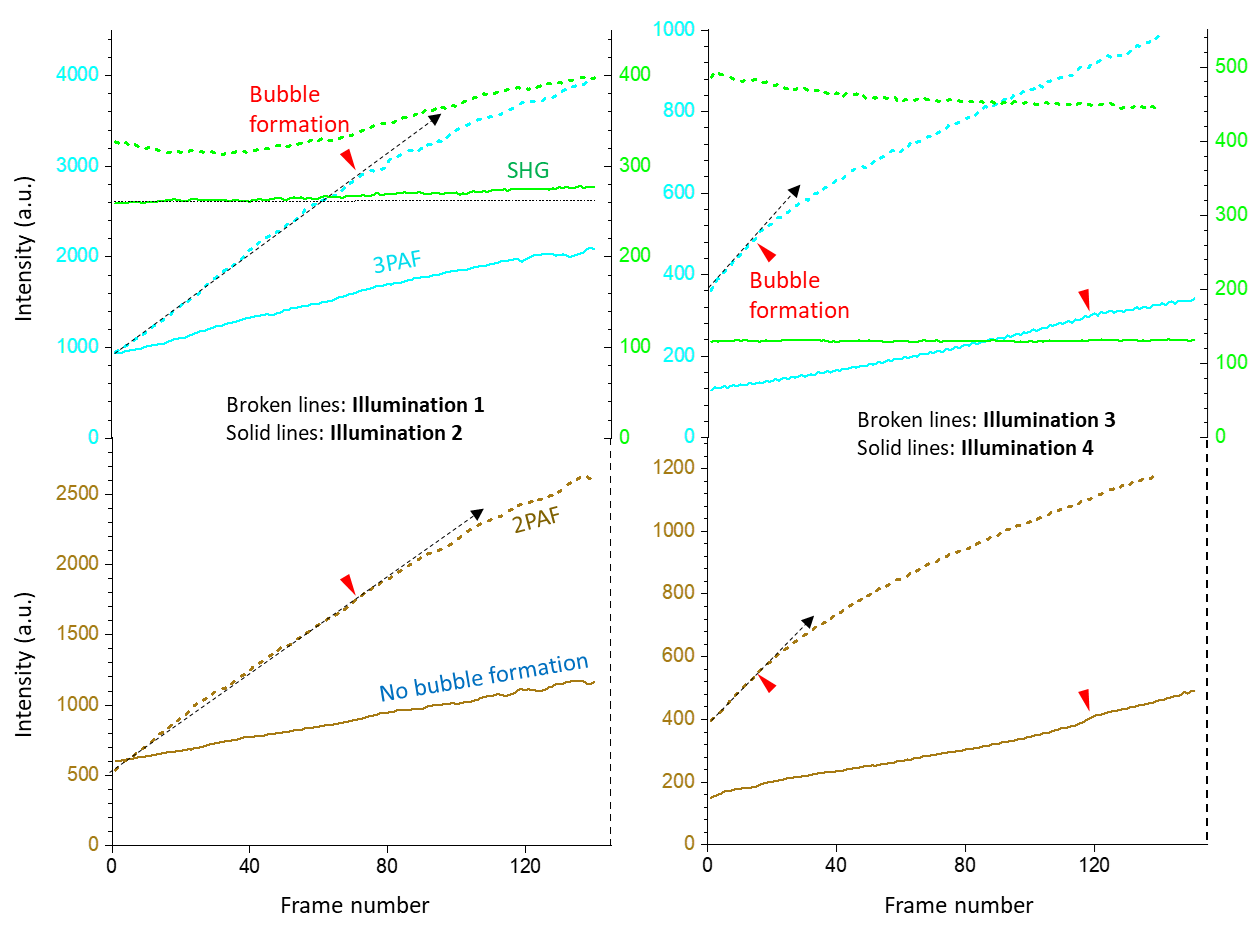
**

**Fig. S6** **Assessment of cavitation (arrowheads) under different pSLAM illuminations.** (Left) cavitation at illumination 1 (0.83 MHz 60 fs, 3.0 mW) absent from illumination 2 at a lower power (0.83 MHz 60 fs, 2.6 mW); (Right): cavitation at illumination 3 (0.83 MHz 300 fs, 5.9 mW) occurs earlies than at illumination 4 (5.0 MHz 300 fs, 20.9 mW) despite the higher power of the latter, indicating the larger role of single-pulse heating than overall thermal load to promote cavitation. Comparison between Illumination 1 and Illumination 3 (or Illumination 2 and Illumination 4) reveals the larger role of photoionization than heating (relevant to cavitation) to accelerate 3PAF/2PAF growth rates (i.e., phototoxicity).


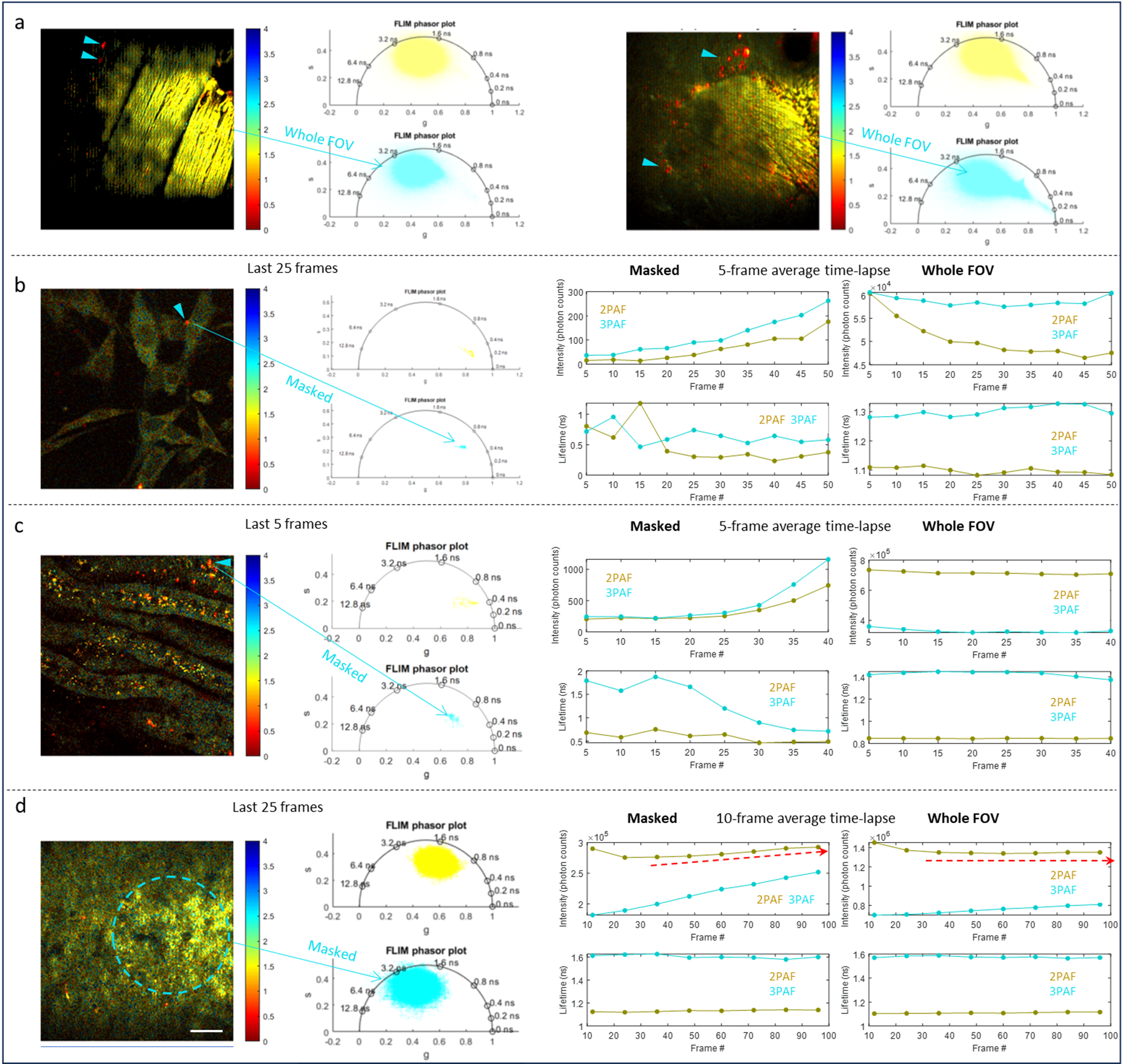


**Fig. S7 FLIM analysis of WHF in cultured cells and *ex vivo* tissue** (color bar corresponds to lifetime in ns). **a** Fluorescence lifetime and phasor plot (FAD – yellow; NADH – cyan) observed from two fresh chicken breast samples with homogeneous WHF (yellow center) and punctuated homogeneous WHF (arrowheads). **b** Heterogeneous WHF lifetime (arrowhead) and phasor plot observed from a hamster kidney cell in cell culture (left two panels) at 18 mW; right panels show time-lapse fluorescence intensity and lifetime corresponding to the heterogeneous WHF only (left) and whole FOV (right). **c** Heterogeneous WHF lifetime (arrowhead) and phasor plot observed from *ex vivo* mouse kidney tissue (left two panels) at 18 mW; right panels show time-lapse fluorescence intensity and lifetime corresponding to the heterogeneous WHF only (left) and whole FOV (right). **d** Homogeneous WHF lifetime (broken cycle) and phasor plot observed from *ex vivo* rat brain slice (left two panels) at 20 mW; right panels show time-lapse fluorescence intensity and lifetime corresponding to the homogeneous WHF only (left) and whole FOV (right) (Video 5). Scale bar: 50 µm.

**
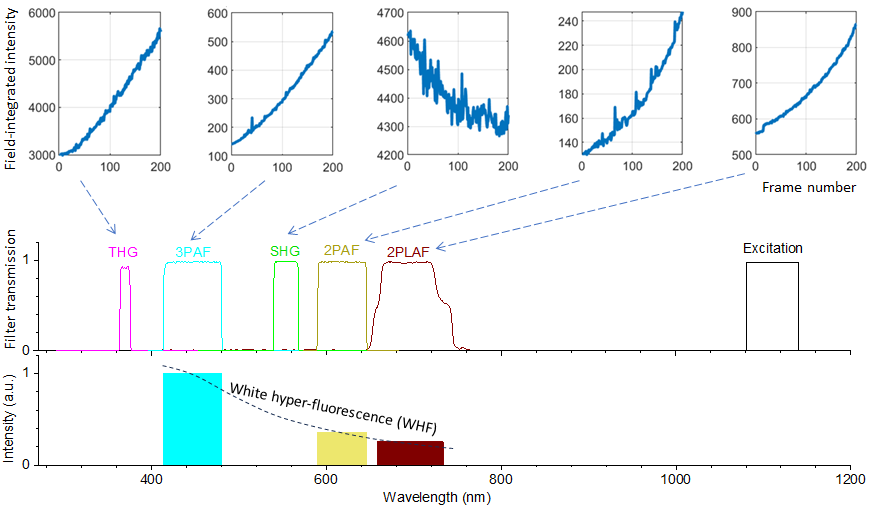
**

**Fig. S8 Detection of white hyper-fluorescence (WHF) in a chicken breast sample across a broad visible range of 420-740 nm.** (Top) Field-integrated intensity versus frame number in time-lapse eSLAM imaging with 5 detection channels; (Middle) Transmission properties of bandpass filters that separate the 5 detection channels into THG, 3PAF, SHG, 2PAF, and 2PLAF; and (Bottom) Normalized intensities of 3PAF, 2PAF, and 2PLAF channels calculated from the top and middle panels based on a calibrated conversion of pixel intensity to photon number, and linked by a broken line that coarsely reveals the spectrum of WHF.

**Table S1. Apparent contradictions in gentle laser-scanning nonlinear optical imaging.**

|  | Popular observation/view | Contradictory observation/view | Observation/view from this study |
| --- | --- | --- | --- |
| Phototoxicity threshold | Absence of a threshold power or pulse energy (irradiance) because phototoxicity depends on fluence or dose**^9,13,44^** | Existence of this threshold below which phototoxicity does not depend on fluence or dose**^8,24,38^** | Unambiguous existence of an irradiance threshold under one pulse per diffraction-limited-resolution imaging |
| Repetition rate (for two-photon imaging) | GHz beneficial**^10,54^** and external pulse splitter are favorable**^11^** | Down to 1-MHz beneficial (even for non-pigmented samples at shallow imaging depths)**^16^** | 5-20 MHz beneficial depending on imaging speed-depth tradeoff (see text in detail) |
| Pulse duration | Irrelevant across a wide regime**^8,26^** (75 fs to 3.2 ps) | Short (<100 fs) pulses beneficial (even for non-pigmented samples)**^31,53,58^** | Short (<100 fs) pulses beneficial due to new hypothesized mechanism (see text) |
| Single-pulse heating | Irrelevant due to low linear absorption of water in non-pigmented samples**^10,34^** and highly nonlinear phototoxicity**^9^** | Relevant (even for non-pigmented samples) from imaging**^33^** to surgery**^35^** | Relevant due to accelerated phototoxicity at low irradiance (phototoxicity) |
| Photo-damage to nucleus | Detectable likely due to ROS-related toxicity with strong hyper-fluorescence**^25,38^** | Not detectable as phototoxic hyper-fluorescence is limited to cytoplasm**^8,24^** | Not likely at the threshold of heating-accelerated phototoxicity |

**Table S2. Dependence of NIR phototoxicity on fast-axis scanning speed or pulses per diffraction-limited-resolution (PPD) of ~80-MHz pulses.**

|  | Slow scanning with high phototoxicity at low irradiance/power/pulse-energy | Fast scanning with low phototoxicity at high irradiance/power/pulse-energy |
| --- | --- | --- |
| Cell viability and WHF assays | Cell death and detectable WHF at **~2 µm/ms** **or ~1.5×10^4^ PPD** (730-nm, 150-fs, 80-MHz, 6 mW)**^38^**, and **~5 µm/ms** **or ~6.2×10^3^ PPD** (780-nm, 170-fs, 80-MHz, 7.3 mW)**^8^** | No cell death (viability assessed by DNA synthesis) and no detectable WHF at **~35 µm/ms or ~860 PPD** (740-nm, 75-fs, 81-MHz, 17 mW)**^13^** |
| Cell ROS assay | ROS production in cultured cells at **~5 µm/ms** **or ~6.4×10^3^ PPD** (800-nm, 170-fs, 80-MHz, 7 mW)**^25^** | No ROS production in developing embryos at **~60 µm/ms** **or ~690 PPD** (1047-nm, 175-fs, 120-MHz, 13-20 mW)**^41,60^** |
| Laser surgery (with WHF) vs. gentle imaging | Laser surgery at **~2 µm/ms** **or ~1.5×10^4^ PPD** (800-nm, ~100-fs, 76-MHz, 60 mW)**^59^** | Gentle imaging at **~2.2×10^4^ µm/ms** **or ~1.4 PPD** (800-nm, ~120-fs, 80-MHz, 200 mW at 250-µm imaging depth)**^33^**, **~2.3×10^3^ µm/ms or ~14 PPD** (720-950-nm, ~100-fs, 80-MHz, 40 mW)**^61^**, and **150 µm/ms or 315 PPD** (1180-nm, 100-fs, 80-MHz, 120 mW)**^39^** |
| Long-term brain calcium imaging | Impossible at <**100 µm/ms** **or >320 PPD** (800-nm, 140-fs, 80-MHz, 18-48 mW)**^62^** | Possible at ~**2.2×10^3^ µm/ms** **or ~14 PPD** (800-nm, 120-fs, 80-MHz, 18 mW)**^62^** |
| Cell functional assays | Functional change at **~24 µm/ms** **or ~1.5×10^3^ PPD** (920-nm, ~100-fs or cw, ~80-MHz, 17 mW at 78-µm imaging depth)**^32^** | No functional change at ~**2.2×10^3^ µm/ms** **or ~14 PPD** (775-nm, 100-ps, 80-MHz, 140 mW)**^18^** |
| CARS imaging | Phototoxicity thresholds at **~50 µm/ms or ~650 PPD** (711/892 nm, 2-ps, 9 mW at 82-MHz)**^44^** | No observable phototoxicity at **~2×10^3^ µm/ms or ~17 PPD** (780-930/1064 nm, ~6-ps, 76-MHz, 100 mW)**^63^** |

Note: Differences in parameters other than fast-axis scanning speed under typical high NA (~1) focusing are ignored; CARS – coherent anti-Stokes Raman scattering microscopy.

**Table S3. Noncomprehensive survey of** **label-free nonlinear optical imaging in biology and medicine.**

| Modality/*Application* | Representative study or review |
| --- | --- |
| Second-harmonic generation microscopy | Campagnola, P.J. & Loew, L.M. Second-harmonic imaging microscopy for visualizing biomolecular arrays in cells, tissues and organisms. *Nat. Biotechnol*. **21**,1356-1360 (2003). |
| Coherent Raman microscopy | Cheng, J.X. & Xie, X.S. Vibrational spectroscopic imaging of living systems: An emerging platform for biology and medicine. *Science* **350**, aaa8870 (2015). |
| Third-harmonic generation microscopy | Weigelin, B., Bakker, G.J. & Friedl, P. Third harmonic generation microscopy of cells and tissue organization. *J. Cell Sci*. **129**, 245-255 (2016). |
| Optical metabolic imaging | Kolenc, O.I. & Quinn, K.P. Evaluating cell metabolism through autofluorescence imaging of NAD(P)H and FAD. *Antioxid. Redox Signal*. **30**, 875-889 (2019). |
| *Preclinical imaging* | Dunn, K.W. et al. Functional studies of the kidney of living animals using multicolor two-photon microscopy. *Am. J. Physiol. Cell Physiol.* **283**, C905-16 (2002). |
| *Developmental biology* | Sun, C.K. et al. Higher harmonic generation microscopy for developmental biology. *J. Struct. Biol.* **147**, 19-30 (2004) |
| *Dermatology* | Chen, S.Y., Wu, H.Y. & Sun, C.K. In vivo harmonic generation biopsy of human skin. *J. Biomed. Opt.* **14**, 060505 (2009). |
| *Drug discovery* | Tipping, W.J., Lee, M., Serrels, A., Brunton, V.G. & Hulme, A.N. Stimulated Raman scattering microscopy: an emerging tool for drug discovery. *Chem. Soc. Rev*. **45**, 2075-2089 (2016). |
| *Histology* | Orringer, D.A. et al. Rapid intraoperative histology of unprocessed surgical specimens via fibre-laser-based stimulated Raman scattering microscopy. *Nat. Biomed. Eng*. **1**, 0027 (2017). |
| *Cell therapy* | Walsh, A.J. et al. Classification of T-cell activation via autofluorescence lifetime imaging. *Nat. Biomed. Eng*. **5**, 77-88 (2021). |
| *Assisted reproductive technology* | Shah, J.S., Venturas, M., Sanchez, T.H., Penzias, A.S., Needleman, D.J. & Sakkas, D. Fluorescence lifetime imaging microscopy (FLIM) detects differences in metabolic signatures between euploid and aneuploidy human blastocysts. *Hum. Reprod*. **37**, 400-410 (2022) |
| *Ophthalmology* | Boguslawski, J. et al. In vivo imaging of the human eye using a 2-photon-excited fluorescence scanning laser ophthalmoscope. *J. Clin. Invest*. **132**, e154218 (2022). |

**Table S4. Hyper-fluorescence-like effect observed with diverse multiphoton illuminations in unlabeled non-pigmented live specimens.**

| Terminology (photon order) | Live specimen of model system | Fast-axis scanning speed on sample (and other parameters) | Observation and description |
| --- | --- | --- | --- |
| Broadband luminescence**^45^**  (**1.1**) | Myelin sheath in spinal tissues from guinea pig | stationary for 22-25 s (704 and 880 nm, 2.5-ps, 3.9-MHz, ~2.4 mW, 60×/1.2-NA) | Photodamage of CARS imaging in point scan mode yields a photon order of 1.1-1.8, which increases with incident power |
| Photoenhancement**^68^** (**1-2**) | Rabbit red blood cells | ~4 µm/ms (810-nm, 14 fs (2.2 mW) or 280 fs (7.3 mW), 86-MHz, 40×/1.1-NA) | Photoenhancement occurs less readily with shorter pulses. The overall fluorescence increase is over 15-fold. |
| Fluorescent scar**^59^** (**2.5**) | Diverse cells and tissue types | ~2 µm/ms (800-nm, ~100-fs, 76-MHz, 60 mW, 25×/0.8-NA) | Wounds as small as 1 μm in diameter 20 μm from the surface are made by multiphoton excitation with characteristic fluorescent scar |
| Hyper-fluorescence**^69^ (>2**) | Mouse gut mucosa | ~8 µm/ms (800-nm, 10/220 fs, 85/80 MHz, 40x/1.2-NA) | Narrow bandwidth laser is preferable to ultrabroadband excitation for autofluorescence-2-photon microscopy |
| Intrinsic indicator**^24^** (**3.17**) | Diverse cells and  tissues | 50 µm/ms (781-nm, 1.2 ps, 80-MHz, 27-52 mW, 32×/0.85-NA) | Photodamage indicator independent of *in vivo* or *ex vivo* tissue state; photon order 3.17 for *ex vivo* brain cryosections |
| Fluorescent lesion**^66^** (**4.28**) | Rat basophilic leukemia cells | ~9 µm/ms (740-nm, 145-fs, 80-MHz, 5-25 mW, 1.3-NA) | The frequency of fluorescent lesion formation increases approximately as the fourth power of the laser intensity |
| Photomodulation**^30,65^** (**6**) | Purified collagen samples | ~5 µm/ms (780-nm, 120-fs, 80-MHz, 6-60 mW, 20x/NA 0.5 or 40x/1.3-NA) | Increased two-photon auto-fluorescence and decreased second harmonic generation similar to 60º C denaturation |

Note: this effect has been observed by different research groups in diverse cells, extracellular matrices, and tissue types, independent of laser pulse widths (fs-ns), excitation wavelengths (throughout near-IR), and imaging acquisition parameters

**Table S5. Comparison of heterogeneous and homogeneous WHF in SLAM-based imaging.**

|  | Heterogeneous WHF | Homogeneous WHF |
| --- | --- | --- |
| Prevalence | Inevitably present in cell culture and live tissue at sufficiently high power/irradiance or dosage | Observable in *ex vivo* chicken breast and rat brain but not cell culture and *ex vivo* mouse kidney |
| Initial photodamage site | Random point(s) within the field-of-view | Center of field-of-view with the highest irradiance |
| Time-lapse morphology | Increase of both intensity and spatial scope in a point-spreading fashion | Uniform elevation across a large area of illumination field |
| Nature of phototoxicity | Dependent on light dosage like the cavitation induced by ultrashort pulses | Dependent on threshold power/irradiance like the bubble formation by ultrashort pulses |
| Time-lapse phototoxicity | Not observable in the first frame but with nonlinear increase over frame/time (Fig. S7b,c) | Observable in the first frame with linear increase over frame/time |
| Stage of phototoxicity | Late stage after onset of homogeneous WHF (chicken breast as example) | Early stage during photobleaching-to-WHF transition (chicken breast and rat brain as examples) |
| Lifetime | Short (~0.6 ns) | Long (>1 ns) |
| Quantitative analysis and modeling of phototoxicity | Unsuitable due to random and point-spreading morphology, dependence on light dosage, and nonlinear increase at a late stage of phototoxicity | Suitable due to homogeneous morphology, dependence on threshold power/irradiance, and linear increase at an early stage of phototoxicity |

**Table S6. Illuminations of pSLAM and eSLAM on chicken breast to quantify phototoxicity.**

| Microscope (optical scanner, control software) | pSLAM (galvo-galvo, ScanImage) | | | | eSLAM (resonant-galvo, LabVIEW) |
| --- | --- | --- | --- | --- | --- |
|  | Baseline/ unchirped | Chirped | Spatial bin-3 | Spatiotemporal bin-10 (or bin-3) |  |
| Central wavelength | 1030 nm | 1030 nm | 1030 nm | 1030 nm | 1110 nm |
| Pulse width on sample (FWHM) | 60 fs | 300 fs | 60 fs | 60 fs | 60 fs unchirped (or 300 fs chirped) |
| Bin: pulses/pixel/frame | 1 | 1 | 3 | 10/3 | 1 |
| Illumination *P* on sample | 2.0 mW | 4.0 mW | 2.0 mW | 20/6.0 mW | 18.7 mW |
| Pule repetition rate | 0.83 MHz | 0.83 MHz | 0.83 MHz | 8.3/2.5 MHz | 5 MHz |
| Fast scan line rate | 340 Hz | 340 Hz | 113 Hz | 340 Hz | 1592 Hz |
| Pixel dwelling time | 1.4 µs | 1.4 µs | 4.2 µs | 1.4 µs | 0.2 µs |
| Exposure/acquisition time per frame | 1.5/1.5 s | 1.5/1.5 s | 4.5/4.5 s | 1.5/1.5 s | 0.33/1.37 s |
| Relevant figure(s) in main text | Figs. 3a,3b,3f | Fig. 3a | Fig. 3b | Fig. 3f | Fig. 3d, top |

Note on common features: imaging depth ~10 µm; frame 1024 pixel × 1024 pixel; field-of-view 300 × 300 µm^2^.

**Table S7. Normalized threshold irradiance of NIR phototoxicity versus number of pulses per diffraction-limited-resolution (PPD).**

| Phototoxicity Assay | Wave-length (nm) | FWHM pulse width (ps) | Pulse repetition rate (MHz) | Average power (mW) | Pulse energy (nJ) | NA of objective | Diffraction-limited resolution (µm) | Fast-axis scanning speed in µm/ms (PPD) | Normalized irradiance* |
| --- | --- | --- | --- | --- | --- | --- | --- | --- | --- |
| Model WHF  (eSLAM) | 1110 | 0.06 | 5.0 | 17 | 3.4 | 1.15 | 0.48 | 1600 (1.5) | 49%** |
| Model WHF  (pSLAM) | 1030 | 0.06 | 0.83 | 1.8 | 2.2 | 1.15 | 0.45 | 290  (1.3) | 41%** |
| Calcium response**^53^** | 1300 | 0.04 | 0.8 | 1.6 | 2.0 | 1.05 | 0.62 | ~600 (~1) | 37% |
| Laser surgery**^59^** | 800 | 0.1 | 76 | 60 | 0.79 | 0.8 | 0.50 | ~2  (~1.5×10^4^) | 8.6% |
| Skin cavitation**^7^** | 780 | 0.1 | 0.01 | 0.0035 | 0.35 | 1.2 | 0.33 | No scanning | 9.4% |
| Retina WHF**^31^** | 750 | 0.075 | 8.0 | 1.8 | 0.23 | 1 | 0.38 | ~200  (15) | 5.5% |
| Phototoxicity indicator**^24^** | 781 | 1.2 | 80 | 52 | 0.65 | 0.7 | 0.56 | ~200  (220) | 0.5% |
| Ca^2+^-indicated stress**^19^** | 775 | 100 | 80 | 140 | 1.75 | 1.4 | 0.28 | ~2.2×10^3^  (~14) | 0.1% |
| Lipid motility**^44^** | 711 | 2 | 1 | 2 | 2 | 1.2 | 0.30 | ~50  (~6) | 3% |
| Myelin WHF**^45^** | ~750 | 2.5 | 7.8 | 8.8 | 1.1 | 1.2 | 0.31 | ~100  (~24) | 1.3% |
| Astrocyte hyperactivity**^32^** | 920 | 0.1 | 80 | 17 | 0.21 | 0.95 | 0.48 | ~24  (~1.5×10^3^) | 2.3% |
| Cell viability**^8^** | 780 | 0.17 | 80 | 7.3 | 0.091 | 1.3 | 0.30 | ~5  ( ~6.2×10^3^) | 1.6% |
| Cell WHF**^38^** | 730 | 0.15 | 80 | 6 | 0.075 | 1.25 | 0.29 | ~2  ( ~1.5×10^4^) | 1.6% |

Note: * - by water optical breakdown across 1030-1110 nm; ** - adjusted for measured M^2^ value of 1.10 for pSLAM laser source or 1.16 for eSLAM laser source. PPD = Pulse repetition rate × Diffraction-limited resolution / Fast-axis scanning speed.

**Table S8. Quantitative phototoxicity bioassays with multiphoton excitation.**

| Nature of bioassay (photon order) | Live specimen of model system | Fast axis scanning speed on sample (and typical other parameters) | Details of bioassay |
| --- | --- | --- | --- |
| Inline labeled**^32^**  (**1**) | Cortical astrocytes in mouse brain slices | ~24 µm/ms (920-nm, ~100-fs or cw, ~80-MHz, 17 mW, 20×/0.95-NA) | Calcium microdomain hyperactivity |
| Offline labeled**^70^**  (**1.19-1.28**) | Drosophila melanogaster | 0 µm/ms (800-nm, 37/100-fs, 1-kHz, irradiance 0.1 TW/cm^2^, no focusing) | TUNEL cell assay in salivary glands superficially located in the larva’s body |
| Inline labeled**^26^**  (**2**) | Neocortical neurons in rat brain slices | ~2 µm/ms (870-nm, 75-fs with 3-7 mW or 3.2-ps with 8-24 mW, ~80-MHz, 60×/0.91-NA) | Basal fluorescence of various Ca^2+^-indicators |
| Offline label-free**^8^**  (**2**) | Chinese hamster ovary cells | ~5 µm/ms (780-nm, 170-fs, 80-MHz, 7.3 mW, 40×/1.3-NA) | Reduced cloning efficiency (clone consists of <8 cells) |
| Inline labeled and offline label-free**^9^**  (**2.5**) | Bovine adrenal chromaffin cells | 25 µm/ms (840-nm, 190-fs, 82-MHz, 7-20 mW, 63×/0.9-NA) | Changes in resting [Ca^2+^] level via FURA-2 and degranulation reaction |
| Offline label-free**^39^**  (**2-3**) | Drosophila embryos | 150 µm/ms (1180-nm, 100-fs, 80-MHz, 120 mW, 20x/0.95-NA) | Embryo survival rate and cellularization speed |
| Offline labeled**^71^**  (**2-3**) | Chinese hamster ovary cells | 8-20 µm/ms (695-810 nm, 130-fs, 80-MHz, 14 mW typically, 40x/0.8-NA) | Damage to nucleus like that from exposure to solar ultraviolet light |

**Table S9. Reinterpretation of observed nonlinear NIR phototoxicity in unlabeled non-pigmented samples.**

|  | Evidence supporting multiphoton-absorption-mediated phototoxicity | Reinterpretation to reconcile with linear-absorption-mediated phenomenologically nonlinear phototoxicity |
| --- | --- | --- |
| Power laws in cell assays (0.7-1.0 µm excitation) | Power- and/or pulse-duration-dependent phototoxic assays attain an apparent multi-photon (≥2) order**^8,9,26^** | Acceleration by photoionization and heating of an otherwise linear phototoxicity attains phenomenologically nonlinear phototoxicity |
| Pulsed versus cw phototoxicity | Absence of phototoxicity when pulsed exaction is switched to cw excitation (with larger powers)**^8,64,65^** | Unlike regular linear phototoxicity**^32,45^**, linear-absorption-mediated (fundamentally linear) phenomenologically nonlinear phototoxicity accelerates with increasing power |
| Linked auto-fluorescence bleaching and lesion | Coincidence of photon order in bleaching and lesion generation (~4) suggests NADH as a photosensitizer**^66^** | Acceleration by photoionization-heating and poor indication of phototoxicity by photo-bleaching result in this effect |
| Cellular ROS production and metabolic change (0.7-1.0 µm excitation) | Similarity to UV-visible-induced ROS and metabolic change suggests multiphoton (rather than single-photon) excitation of intrinsic UV-visible sensitizers**^25,38^** | Single-photon excitation of NIR intrinsic photosensitizers with efficient intersystem crossing to triplet state induces this ROS production and metabolic change alternatively |
| Confined phototoxicity in one illumination plane (1.0-1.2 µm excitation) | More phototoxicity from single-plane illumination over multiplane illumination of developing embryos suggests a multiphoton origin**^39^** | Linear-absorption-mediated phenomenologically nonlinear phototoxicity attains an apparent nonlinearity to confine phototoxicity in one illumination plane |
| Water absorption in a high NA microscope objective | Low and wavelength-dependent water absorption prohibits related heating as source for phototoxicity**^10,33^** | Absence of heating via water absorption as the primary source for phototoxicity does not rule out linear phototoxicity from a non-water NIR photosensitizer in the hypothesized mechanism |
| Nonlinear fluorescence photo-bleaching | Fluorescence bleaching attains a >2 photon order (higher than that of “photo-enhancement”)**^67,68^** | Photo-bleaching of intrinsic and extrinsic fluorophores is a poor indicator of phototoxicity**^2,3^** |

**References (continued)**

1. Sibai, M., Mehidine, H., Poulon, F., Ibrahim, A., Varlet, P., Juchaux, M., Pallud, J., Devaux, B., Kudlinski, A. & Haidar, D. A. The impact of compressed femtosecond laser pulse durations on neuronal tissue used for two-photon excitation through an endoscope. *Sci. Rep*. **8**, 11124 (2018).
2. Galbraith, J. A. & Terasaki, M. Controlled damage in thick specimens by multiphoton excitation. *Mol. Biol. Cell* **14**, 1808-1817 (2003).
3. Hockberger, P. E., Skimina, T. A., Centonze, V. E., Lavin, C., Chu, S., Dadras, S., Reddy, J. K. & White, J. G. Activation of flavin-containing oxidases underlies light-induced production of H_2_O_2_ in mammalian cells. *Proc. Nat. Acad. Sci. USA* **96**, 6255-6260 (1999).
4. Lee, A. M. D., Wang, H., Yu, Y., Tang, S., Zhao, J., Lui, H., McLean, D. I. & Zeng, H. *In vivo* video rate multiphoton microscopy imaging of human skin. *Opt. Lett.* **36**, 2865-2867 (2011).
5. Chen, X., Leischner, U., Varga, Z., Jia, H., Deca, D., Rochefort, N. L. & Konnerth, A. LOTOS-based two-photon calcium imaging of dendritic spines in vivo. *Nat. Protoc.* **7**, 1818-1829 (2012).
6. Evans, C. L., Potma, E. O., Puoris'haag, M., Côté, D., Lin, C. P. & Xie, X. S. Chemical imaging of tissue *in vivo* with video-rate coherent anti-Stokes Raman scattering microscopy. *Proc. Nat. Acad. Sci. USA* **102**, 16807-16812 (2005).
7. König, K., Liang, H., Berns, M. W. & Tromberg, B. J. Cell damage in near-infrared multimode optical traps as a result of multiphoton absorption. *Opt. Lett.* **21**, 1090-1092 (1996).
8. Hovhannisyan, V., Ghazaryan, A., Chen, Y. F., Chen, S. J. & Dong, C. Y. Photophysical mechanisms of collagen modification by 80 MHz femtosecond laser. *Opt. Express* **18**, 24037-24047 (2010).
9. Tiede, L. M. & Nichols, M. G. Photobleaching of reduced nicotinamide adenine dinucleotide and the development of highly fluorescent lesions in rat basophilic leukemia cells during multiphoton microscopy. *Photochem. Photobiol.* **82**, 656-664 (2006).
10. Patterson, G. H. & Piston, D. W. Photobleaching in two-photon excitation microscopy. *Biophys. J.* **78**, 2159-2162 (2000).
11. Pestov, D., Andegeko, Y., Lozovoy, V. V. & Dantus, M. Photobleaching and photoenhancement of endogenous fluorescence observed in two-photon microscopy with broadband laser sources. *J. Opt.* **12**, 084006 (2010).
12. Klinger, A., Krapf, L., Orzekowsky-Schroeder, R., Koop, N., Vogel, A. & Hüttmann, G. Intravital autofluorescence 2-photon microscopy of murine intestinal mucosa with ultra-broadband femtosecond laser pulse excitation: image quality, photodamage, and inflammation. *J. Biomed. Opt.* **20**, 116001 (2015).
13. Saytashev, I., Arkhipov, S. N., Winkler, N., Zuraski, K., Lozovoy, V. V. & Dantus, M. Pulse duration and energy dependence of photodamage and lethality induced by femtosecond near infrared laser pulses in Drosophila melanogaster. *J. Photochem. Photobio. B.* **115**, 42-50 (2012).
14. Nadiarnykh, O., Thomas, G., Van Voskuilen, J., Sterenborg, H. J. & Gerritsen, H. C. Carcinogenic damage to deoxyribonucleic acid is induced by near-infrared laser pulses in multiphoton microscopy via combination of two-and three-photon absorption. *J. Biomed. Opt.* **17**, 116024 (2012).

**Video 1.** Time-lapse eSLAM imaging of hamster kidney cells showing heterogeneous WHF (arrowheads in Fig. 2a).

**Video 2**. Time-lapse eSLAM imaging of *ex vivo* mouse kidney tissue showing heterogeneous WHF (arrowheads in Fig. 2b).

**Video 3**. Time-lapse pSLAM imaging of chicken breast showing early homogeneous WHF versus late heterogeneous WHF and cavitation (see also in Fig. 2c; SHG – green, 3PAF – cyan, 2PAF – yellow).

**Video 4**. Time-lapse eSLAM imaging (15 mW on sample) of mouse red blood cells showing homogeneous WHF across the field-of-view.

**Video 5**. Time-lapse eSLAM imaging of *ex vivo* rat brain slice showing homogeneous WHF via 3PAF (cyan) but not 2PAF (yellow) or THG (magenta).

**Video 6**. Time-lapse eSLAM imaging of moving *C. elegans* worms.
